# Circular RNA circ_001422 promotes the progression and metastasis of osteosarcoma via the miR-195-5p/FGF2/PI3K/Akt axis

**DOI:** 10.1186/s13046-021-02027-0

**Published:** 2021-07-16

**Authors:** Bingsheng Yang, Lutao Li, Ge Tong, Zhirui Zeng, Jianye Tan, Zexin Su, Zhengwei Liu, Jiezhao Lin, Wenwen Gao, Jianping Chen, Sisi Zeng, Guofeng Wu, Lin Li, Shuang Zhu, Qiuzhen Liu, Lijun Lin

**Affiliations:** 1 Department of Joint and Orthopedics, Zhujiang Hospital, Southern Medical University, Guangzhou, 510282 China; 2 Department of Medical Ultrasonics, Guangdong Province Key Laboratory of Hepatology Research, The Third Affiliated Hospital of Sun Yat-Sen University, Guangzhou, 510630 China; 3 Guizhou Provincial Key Laboratory of Pathogenesis & Drug Research on Common Chronic Diseases, Department of Physiology, School of Basic Medicine, Guizhou Medical University, Guiyang, 550009 China; 4 Department of Musculoskeletal Oncology, The First Affiliated Hospital of Sun Yat-Sen University, Guangzhou, 510080 China; 5 Department of Spinal Surgery, Zhujiang Hospital, Southern Medical University, Guangzhou, 510282 China; 6 Guangdong Provincial Key Laboratory of Cancer Immunotherapy, Guangzhou Key Laboratory of Tumor Immunology Research, Cancer Research Institute, School of Basic Medical Sciences, Southern Medical University, Guangzhou, 510515 China

**Keywords:** Osteosarcoma, Circ_001422, miR-195-5p, FGF2, PI3K/Akt pathway

## Abstract

**Background:**

Circular RNAs (circRNAs) are involved in diverse processes that drive cancer development. However, the expression landscape and mechanistic function of circRNAs in osteosarcoma (OS) remain to be studied.

**Methods:**

Bioinformatic analysis and high-throughput RNA sequencing tools were employed to identify differentially expressed circRNAs between OS and adjacent noncancerous tissues. The expression level of circ_001422 in clinical specimens and cell lines was measured using qRT-PCR. The association of circ_001422 expression with the clinicopathologic features of 55 recruited patients with OS was analyzed. Loss- and gain-of-function experiments were conducted to explore the role of circ_001422 in OS cells. RNA immunoprecipitation, fluorescence in situ hybridization, bioinformatics database analysis, RNA pulldown assays, dual-luciferase reporter assays, mRNA sequencing, and rescue experiments were conducted to decipher the competitive endogenous RNA regulatory network controlled by circ_001422.

**Results:**

We characterized a novel and abundant circRNA, circ_001422, that promoted OS progression. Circ_001422 expression was dramatically increased in OS cell lines and tissues compared with noncancerous samples. Higher circ_001422 expression correlated with more advanced clinical stage, larger tumor size, higher incidence of distant metastases and poorer overall survival in OS patients. Circ_001422 knockdown markedly repressed the proliferation and metastasis and promoted the apoptosis of OS cells in vivo and in vitro, whereas circ_001422 overexpression exerted the opposite effects. Mechanistically, competitive interactions between circ_001422 and miR-195-5p elevated FGF2 expression while also initiating PI3K/Akt signaling. These events enhanced the malignant characteristics of OS cells.

**Conclusions:**

Circ_001422 accelerates OS tumorigenesis and metastasis by modulating the miR-195-5p/FGF2/PI3K/Akt axis, implying that circ_001422 can be therapeutically targeted to treat OS.

**Supplementary Information:**

The online version contains supplementary material available at 10.1186/s13046-021-02027-0.

## Background

Osteosarcoma (OS) is the most prevalent primary malignant bone neoplasm causing substantial morbidity in adolescents and children [1]. It originates from mesenchymal cells and is characterized by rapid infiltrating growth, early lung metastasis and a high recurrence rate [2]. Studies have shown that the overall 5-year survival rate of patients with localized OS ranges between 65 and 75% and is only 20% for those with recurrent and metastatic tumors [3]. Despite advances in OS treatment approaches such as adjuvant chemotherapy and surgical resection, the survival rates have plateaued in the last 3 decades and are less than satisfactory [4]. Indeed, no specific diagnostic and prognostic biomarkers for OS have been found. Consequently, molecular studies aiming to identify promising therapeutic targets for OS are urgently needed.

Circular RNAs (circRNAs) regulate various functions of eukaryotic cells [5]. Based on the order of splicing events and different intermediates, two mechanisms exist for the biogenesis of circRNAs: canonical spliceosome-induced splicing and noncanonical lariat splicing [6, 7]. Accumulating studies have shown that circRNAs modulate diverse physiological and pathophysiological processes by sponging microRNAs (miRNAs), interacting with RNA-binding proteins, and modulating epigenetic, transcriptional, or translational alterations in target genes [8–11]. Abnormal circRNA expression has been found to correlate with the pathogenesis of various cancers and to exert essential regulatory effects on gene expression, cell invasion, cell cycle progression, migration, apoptosis, and proliferation [12–14]. Moreover, circRNAs are thought to possess high diagnostic and therapeutic potential given their structural stability, evolutionary conservation, abundance and organ specificity [15, 16]. However, to date, the roles of circRNAs in OS are not clearly known.

This study evaluated the expression profiles of circRNAs in OS tissues and adjacent noncancerous tissues using high-throughput sequencing. We found a novel circRNA, designated circ_001422, that regulates the progression of OS. Higher expression of circ_001422 was markedly associated with more advanced clinical stage, large tumor size, higher incidence of metastases and poorer prognosis. Experimental results indicated that circ_001422 exerted pro-oncogenic effects on OS proliferation and metastasis by targeting the miR-195-5p/FGF2/PI3K/Akt axis. Our findings revealed that circ_001422 is a potential therapeutic target for OS.

## Methods

### Collection of patient samples

This study was approved by the Ethics Committee of the Affiliated Zhujiang Hospital of Southern Medical University (approval no. 2018-GJGBWK-002) and conducted in accordance with the Declaration of Helsinki. A total of 55 patients with OS were enrolled in the study between May 2018 and April 2020. All participants underwent diagnostic core needle biopsy using a disposable sterile biopsy instrument (Trauson Medical Instrument Corporation, China). Histological diagnosis was performed independently by two experienced pathologists. Written informed consent was obtained from all participants and, for minors, from their legal guardian. Surgically removed OS tissues and adjacent noncancerous tissues were collected from these patients before the commencement of chemotherapy, radiotherapy or immunotherapy. Morphologically normal muscle tissues that were more than 5 cm from the cancerous tissues were used as adjacent noncancerous tissues. Upon resection, tissues were immediately frozen in liquid nitrogen and stored at − 80 °C. Table 1 shows the patients’ clinical characteristics.

**Table 1:** Correlations between circ_001422 expression and clinicopathological characteristics of patients with OS

Parameters	Group	Cases	RQ value of circ_001422	*P* value
Age (years)				0.520
	≤ 18	30	3.108 ± 1.529	
	> 18	25	3.373 ± 1.494	
Gender				0.150
	Male	32	3.477 ± 1.579	
	Female	23	2.882 ± 1.354	
Clinical stage				0.019
	I	19	2.801 ± 1.528	
	II	20	2.938 ± 1.067	
	III-IV	16	4.098 ± 1.661	
Tumor size (cm)				0.003
	≤ 5	24	2.557 ± 0.881	
	> 5	31	3.748 ± 1.686	
Distant metastasis				0.005
	Absent	39	2.871 ± 1.296	
	Present	16	4.098 ± 1.661	
Primary tumor location				0.818
	Arm/hand	20	3.381 ± 1.591	
	Leg/foot	31	3.168 ± 1.539	
	Others	4	2.929 ± 0.850	

Relative quantification (RQ) values of circ_001422 are presented as means ± standard deviations

### Cell culture

A human osteoblast cell line (hFOB1.19) and OS cell lines (143B, U-2 OS, MG-63, MNNG and Saos-2) were purchased from Jennio (Guangzhou, China). Osteoblastic hFOB1.19 cells were cultured in DMEM/F-12 (Gibco, USA) supplemented with 10% fetal bovine serum (FBS) (BI, Israel), 2.5 mM L-glutamine (Invitrogen, USA) and 0.3 mg/ml geneticin (Gibco, USA). OS cells were maintained in DMEM supplemented with 1% penicillin/streptomycin (Invitrogen, USA) and 10% FBS in a humidified incubator at 37 °C in 5% CO_2_.

### RNA sequencing (RNA-seq)

High-throughput sequencing was performed to identify circRNAs through the following steps. Total cellular RNA was isolated from 3 matched OS tissues and adjacent noncancerous tissues with an RNAiso Plus Reagent Kit (TaKaRa, Japan). Ribosomal RNA was removed with a Ribo-Zero Magnetic Kit (Epicentre, USA), whereas linear RNA was digested with RNase R (Epicentre, USA). Ribosomal RNA depletion and total RNA quality were assessed using a TapeStation 2200 system (Agilent Technologies, USA) and a Qubit RNA high-sensitivity fluorimeter (Thermo Fisher Scientific, USA). Fragmentation was carried out using divalent cations in an Ambion proprietary fragmentation buffer at 94 °C for 5 min and was followed by ethanol precipitation. The RNA fragments were resuspended in nuclease-free water and purified using Agencourt RNA Clean XP Beads (Beckman Coulter, USA). First-strand complementary DNA (cDNA) was synthesized using random hexamer primers and SuperScript II reverse transcriptase (Thermo Fisher Scientific, USA) with the following thermal cycling conditions: 25 °C for 10 min, 42 °C for 15 min, and 70 °C for 15 min. Subsequently, second-strand cDNA synthesis was performed at 16 °C for 1 h after the addition of second-strand synthesis reaction buffer, dNTPs, RNase H, and DNA polymerase I. Double-stranded cDNA was purified using 1.8× Agencourt AMPure XP Beads (Beckman Coulter, USA) and was then subjected to end repair, 3′ adenylation and adaptor ligation by using an NEBNext Ultra II DNA Library Prep Kit for Illumina (New England Biolabs, USA). To select cDNA fragments of the preferred length (200–300 bp), the ligation reaction product was treated with USERTM enzyme at 37 °C for 15 min and purified using Agencourt AMPure XP Beads. PCR was carried out by an initial denaturation step for 30 s at 98 °C followed by 12 cycles of denaturation for 10 s at 98 °C, annealing for 75 s at 65 °C, and extension for 5 min at 65 °C. PCR products were purified with 1× Agencourt AMPure XP beads and subjected to quality control using a High Sensitivity DNA Assay Kit (Agilent Technologies, USA). After quantification and pooling in a StepOnePlus Real-Time PCR System (Applied Biosystems, USA), the cDNA libraries were sequenced on the Illumina HiSeq 2500 platform by Gene Denovo Biotechnology (Guangzhou, China) according to the manufacturer’s instructions. Raw reads in FASTQ format were analyzed and preprocessed using fastp software [17] (version 0.19.1) to remove reads containing adaptors with more than 10% unknown nucleotides or more than 50% low-quality (Q-value ≤10) bases. The remaining high-quality clean reads were aligned to the *Homo sapiens* ribosomal RNA database and the human GRCh38 reference genome using Bowtie2 [18] (version 2.2.8) and TopHat2 [19] (version 2.1.1). The unmapped reads were extracted and processed with find_circ [20] (version 1) for circRNA identification. To quantify the expression levels of the circRNAs, the back-spliced junction reads were scaled to reads per million mapped reads (RPM). Differentially expressed circRNAs with | fold change | ≥ 2 and *P* value < 0.05 were identified using the limma package (version 3.42.0) (bioconductor.org/packages/release/bioc/html/limma.html).

### Measurement of RNA expression

Total RNA was extracted from cultured cells or clinical tissues using an RNAiso Plus Reagent Kit (TaKaRa, Japan) according to the manufacturer’s instructions. RNA integrity was confirmed through agarose gel electrophoresis, and RNA concentration and purity were determined with a Nanodrop 2000 spectrophotometer (Thermo Fisher Scientific, USA). Final RNA quality was assessed in an Agilent 2100 Bioanalyzer (Agilent Technologies, USA), and a minimal RNA integrity number (RIN) of 8 was required. For quantitative analysis of circRNAs and mRNAs, cDNA was synthesized with a PrimeScript RT Reagent Kit (TaKaRa, Japan). Real-time amplification was conducted using SYBR Premix Ex Taq II (TaKaRa, Japan) in a LightCycler 96 System (Roche, Germany). For miRNA analysis, reverse transcription was performed using stem-loop RT primers specific for the miRNA of interest (GeneChem, China) based on an Evo M-MLV RT Kit (Accurate Biology, China), and qPCR was then performed with a SYBR Green Premix Pro Taq HS qPCR Kit (Accurate Biology, China). The following thermal cycling program was used for qPCR of all RNAs: denaturation at 95 °C for 30 s followed by 45 cycles of denaturation at 95 °C for 5 s, annealing at 55 °C for 30 s, and extension at 72 °C for 30 s. GAPDH (for circRNAs and mRNAs) and U6 (for miRNAs) were used as the endogenous controls. Relative quantification (RQ) values were calculated using the following equation: RQ = 2^−ΔΔCt^, where ΔΔCt = [Ct (gene of interest, sample) - Ct (GAPDH or U6, sample)] - [Ct (gene of interest, calibrator) - Ct (GAPDH or U6, calibrator)]. All experiments were performed independently at least three times, and all samples were analyzed in triplicate. Primer sequences are provided in Additional file 1: Table S1.

### RNase R digestion, nucleic acid electrophoresis and Sanger sequencing

Total RNA (2 μg) was extracted and digested with RNase R (3 U/μg) for 15 min at 37 °C. The control samples were processed in the same way as the experimental samples except that RNase R was not added. cDNA synthesis and real-time PCR were performed as described above. Additionally, circular and linear transcripts were amplified using specific divergent and convergent primers with or without RNase R. The PCR products amplified from cDNA or genomic DNA (gDNA) templates were added to 6× loading buffer with the nucleic acid dye GelRed (Biotium, USA). DNA fragments were separated by agarose gel (2%) electrophoresis at 100 V for 30 min and visualized with UV transillumination. For Sanger sequencing, PCR amplification products were excised from the agarose gel and purified using a GeneJET Gel Purification Kit (Thermo Fisher Scientific, USA). The nucleotide sequences of the purified fragments were determined by Sanger sequencing using standard approaches [21] by Geneseed (Guangzhou, China).

### Transcriptional inhibition assay with actinomycin D

In brief, OS cells were cultured in six-well plates for 24 h, and fresh medium supplemented with 2 μg/ml actinomycin D (Sigma-Aldrich, USA) was then added. Total cellular RNA was extracted 0, 4, 8, 12 and 24 h after actinomycin D treatment for qRT-PCR analysis.

### Nucleocytoplasmic fractionation

Extraction and purification of cytoplasmic and nuclear RNAs were performed with a PARIS kit (Life Technologies, USA) in accordance with the manufacturer’s protocols. Next, qRT-PCR was performed to quantify the expression of linear RNAs and circRNAs, with U6 and GAPDH as the internal references for nuclear and cytoplasmic RNAs, respectively.

### Fluorescence in situ hybridization (FISH)

RNA-FISH was performed to determine the subcellular localization of circ_001422. The Cy3-labeled circ_001422 probe was constructed by RiboBio (Guangzhou, China). Fluorescence signals were generated using a Fluorescence In Situ Hybridization Kit (RiboBio, China), and a Nikon A1 confocal laser scanning microscope (Nikon, Japan) was utilized to take pictures.

### Oligonucleotides, plasmids, cell transfection and lentiviral transduction

The miR-195-5p mimic/inhibitor, miR-195-5p agomir/antagomir and their corresponding negative controls (NC) were purchased from RiboBio (Guangzhou, China). For construction of knockdown plasmids expressing short hairpin RNAs (shRNAs) against circ_001422 or FGF2, the pLshRNA-NC and pLKO.1 vectors were constructed by Geneseed (Guangzhou, China), and the annealed shRNA oligonucleotides targeting circ_001422 or FGF2, respectively, were ligated into these vectors. Additionally, the full-length cDNA sequences of circ_001422 and FGF2 were PCR amplified and cloned into the pLC5-ciR vector (Geneseed, China) or pcDNA3.1 vector (Geneseed, China), respectively, to construct the circ_001422 and FGF2 overexpression plasmids. The nontargeting pLC5-ciR and pcDNA empty vectors were used as the corresponding negative controls. 293 T packaging cells were transiently transfected with the abovementioned plasmids using EndoFectin Max reagent (GeneCopoeia, USA) for production of lentiviral particles. The viral supernatants were collected and concentrated using a Lenti-X Concentrator Kit (Clontech, USA). All lentiviral particles in this study had a titer of 1 × 10^9^ transducing units/ml and were stored at − 80 °C. Finally, the concentrated lentivirus was used to transduce 143B and Saos-2 cell lines in logarithmic growth phase. At 48 h post lentiviral transduction, OS cells were selected with puromycin (2 μg/ml, Invitrogen, USA) or geneticin (500 μg/ml, Gibco, USA) for 2 weeks to obtain stable cell lines. The knockdown or overexpression efficiency was verified using qRT-PCR. Notably, the OS cells transduced with sh-circ_001422#2 or sh-FGF2#1 showed the lowest transcription level of the corresponding target gene, and were therefore used in subsequent experiments.

### Cell proliferation assays

The viability of 143B and Saos-2 cells after transfection was assessed using 5-ethynyl-2′-deoxyuridine (EdU) incorporation and colony formation assays. An EdU Apollo 488 Kit (RiboBio, China) was utilized to conduct the EdU incorporation assay. For evaluation with a Nikon inverted fluorescence microscope, EdU-positive cells were stained green, and nuclei were stained blue.

To evaluate the colony-forming ability of cells, transfected cells were counted and seeded into 6-well plates at 550 cells/well. Cells were cultured in complete DMEM supplemented with 10% FBS at 37 °C and 5% CO_2_. The culture medium was replaced at 2-day intervals. After 10 days of incubation, the colonies were washed twice with phosphate-buffered saline (PBS) and fixed with 4% paraformaldehyde for 20 min. Then, the stationary liquid was removed, and 0.1% crystal violet (Solarbio, China) was added for 20 min of staining. The 6-well plates were gently rinsed with water, and colonies with > 50 cells were counted under an optical microscope (Olympus, Japan).

### Flow cytometry

The cell cycle distribution of transfected cells was assessed by the following procedure. In brief, cultured cells were harvested, washed twice in PBS and fixed overnight at 4 °C with precooled 75% ethanol. After staining with propidium iodide, the cell cycle distribution was analyzed with a BD flow cytometer. 4′,6-Diamidino-2-phenylindole (DAPI) and Annexin-V-allophycocyanin (APC) double staining kits (BestBio, China) were used for apoptosis analyses.

### Transwell assays

The migration and invasion abilities of OS cell lines were evaluated using Transwell migration chambers (Costar, USA) and Transwell invasion chambers precoated with 50 μl of 2 mg/ml Matrigel (BD Biosciences, USA), respectively. In brief, transfected cells (4 × 10^4^ cells/well for the migration assay, 8 × 10^4^ cells/well for the invasion assay) suspended in 200 μl of serum-free DMEM were seeded into the upper chambers. A 600 μl volume of DMEM supplemented with 10% FBS was used as the attractant and was added into the lower chambers. After culture for 24 h, cells adhering to the lower surface of the membrane were fixed with paraformaldehyde (4%) and stained using crystal violet (0.1%), whereas cells on the upper surface of the membrane were removed by wiping with cotton swabs. At least three random fields of view containing cells that had migrated or invaded to the lower surface were imaged under an inverted light microscope.

### Western blot analysis

Total protein from homogenized tissues or cell lysates was extracted using ice-cold RIPA solution (Fudebio, China) and protease inhibitors (Fudebio, China). The protein samples were diluted to equal concentrations, denatured in a boiling water bath, separated by SDS-PAGE and transferred onto polyvinylidene difluoride membranes. Membranes were blocked with 5% nonfat milk in Tris-buffered saline-Tween (TBST) buffer and were then incubated overnight at 4 °C with primary antibodies against cleaved CASP3 (1:1000) (Affinity Biosciences, USA), CCND1 (1:1200) (Proteintech, China), CDK4 (1:2000) (Abcam, USA), BAX (1:1000) (Abcam, USA), BCL2 (1:800) (Abcam, USA), E-cadherin (1:2000) (Proteintech, China), N-cadherin (1:2000) (Proteintech, China), Vimentin (1:3000) (Proteintech, China), FGF2 (1:200) (Santa Cruz Biotechnology, USA), PI3K (1:1000) (Cell Signaling Technology, USA), phosphorylated PI3K (p-PI3K, 1:1000) (Cell Signaling Technology, USA), Akt (1:1000) (Cell Signaling Technology, USA), phosphorylated Akt (p-Akt, 1:1000) (Cell Signaling Technology, USA) and GAPDH (1:10000) (Proteintech, China). After four washes with TBST buffer, membranes were incubated for 60 min at 25 °C with horseradish peroxidase (HRP)-conjugated secondary antibodies (1:10000) (Bioss, China). Protein bands were visualized with a chemiluminescence imaging system (Bio-Rad, USA).

### Animal models

The protocols for animal experiments were approved by the Medical Ethics Committee of Southern Medical University. Four-week-old female BALB/c athymic nude mice were purchased from Guangdong Medical Laboratory Animal Center (Guangdong, China) and reared in a pathogen-free facility with access to adequate standard food and water. The subcutaneous xenograft model was established by subcutaneous injection of 5 × 10^6^ stable 143B cells suspended in 100 μl of PBS into the right limbs of nude mice (*n* = 5 mice per group). Tumors were measured every 3 days, and tumor volumes were calculated using the following equation: volume = length × width^2^ × 0.5. To establish the lung metastasis model, the abovementioned cells were injected into nude mice via the tail vein (2 × 10^6^ 143B cells in 200 μl of PBS per mouse, *n =* 5 mice per group) to mimic tumor metastasis. To evaluate the effect of miR-195-5p in vivo, the miR-195-5p antagomir/agomir or corresponding negative control was injected intratumorally (for the subcutaneous xenograft tumor model) or intravenously via the tail vein (for the lung metastasis model) twice weekly for 2 weeks in accordance with the manufacturer’s recommendations (RiboBio, China). Tumor tissues were extracted from sacrificed mice 4 weeks after inoculation. All mice were euthanized by CO_2_ asphyxiation in accordance with the American Veterinary Medical Association (AVMA) Guidelines for the Euthanasia of Animals (2013 Report of the AVMA Panel of Euthanasia).

### Hematoxylin and eosin (H&E) staining

Paraffin-embedded thin (4-μm) lung sections containing metastatic nodules were dewaxed using xylene and were then rehydrated through an alcohol gradient. Then, sections were stained using H&E for general histological examination by standard procedures.

### Assessment of tissue expression of target proteins

The expression of target proteins in tissue samples from OS patients or animal xenograft models was determined using immunohistochemistry (IHC) as previously described [22]. Tissues were incubated with primary antibodies against FGF2 (1:100), Ki-67 (1:200), PCNA (1:300), N-cadherin (1:200), E-cadherin (1:200), and Vimentin (1:300). Except for the anti-FGF2 antibody (Santa Cruz Biotechnology, USA), all antibodies were purchased from Proteintech (China). After washing, the tissue sections were incubated with HRP-conjugated secondary antibodies (1:200) (Servicebio, China) and were then stained with diaminobenzidine (Zhongshan Golden Bridge, China). The tissues were observed and imaged using an optical microscope (Olympus, Japan).

### TUNEL assay

To assess apoptotic DNA fragmentation, xenograft tumor tissues were first fixed for 24 h with 4% paraformaldehyde and were then embedded in paraffin. Apoptosis in situ was evaluated with a TUNEL Apoptosis Assay Kit (Alexa Fluor 488) (Yeasen, China). Corresponding images of the apoptotic cells were acquired using a fluorescence microscope. Analyses were performed with at least three random fields of view per sample.

### Bioinformatic analysis

To predict the potential miRNAs binding with circ_001422, five online bioinformatics tools were used: starBase (http://starbase.sysu.edu.cn/), miRanda (http://www.microrna.org/), TargetScan (http://www.targetscan.org/vert_72/), RNAhybrid (http://bibiserv.techfak.uni-bielefeld.de/rnahybrid/), and RNA22 (https://cm.jefferson.edu/rna22/). The potential downstream target mRNAs of miR-195-5p were predicted after analysis with the miRanda, RNAhybrid, TargetScan and miRTarBase (http://mirtarbase.mbc.nctu.edu.tw/) databases.

### RNA immunoprecipitation (RIP)

Transfected cells were washed twice in ice-cold PBS and were then lysed in RIP lysis solution containing RNase and protease inhibitors. Cell lysates (200 μl) were incubated overnight at 4 °C with immunoprecipitation buffer containing anti-Argonaute2 (anti-Ago2)-conjugated magnetic beads (Millipore, USA) or negative control anti-IgG (Millipore, USA). Subsequently, the immunoprecipitated RNAs were extracted and purified to determine the abundances of the target RNAs by qRT-PCR.

### RNA pulldown assay

A control probe and a biotinylated circ_001422 probe were constructed by RiboBio (Guangzhou, China). The probes were coated with C-1 magnetic beads (Life Technologies, USA) after incubation for 2 h at room temperature with the abovementioned beads. Transfected cells were harvested and treated with ice-cold lysis solution and were then incubated overnight at 4 °C with the circ_001422 or oligo probes. Finally, the precipitates were extracted and purified using an RNeasy Mini Kit (Qiagen, USA). The abundances of circ_001422 and miRNAs in the RNA complexes were evaluated by qRT-PCR.

### Luciferase reporter assay

The circ_001422 or FGF2 fragments with the mutant (MUT) or wild-type (WT) miR-195-5p binding sites were subcloned downstream of the Renilla gene in the psiCHECK-2 dual-luciferase reporter vector (Geneseed, China). 143B and Saos-2 cells in logarithmic growth phase were cotransfected with the reporter vectors and the miR-195-5p mimic or NC mimic. After 48 h of incubation, a Dual-Luciferase Reporter Assay System (Promega, USA) was utilized to measure luciferase activity.

### Statistical analysis

Data for continuous variables are presented as means ± standard deviations. All analyses were performed using SPSS 20.0 software (IBM, USA). All experiments were performed with three technical replicates, and at least three biological replicates were performed. Differences between groups were analyzed using unpaired Student’s t-test or one-way analysis of variance (ANOVA) with Tukey’s test. Survival was analyzed by the Kaplan-Meier method and the log-rank test. Correlations among the levels of circ_001422, miR-195-5p and FGF2 were evaluated using Pearson correlation analysis. *P* < 0.05 was considered to indicate a significant difference.

## Results

### Identification and characterization of circ_001422 in OS

**Fig. 1:**
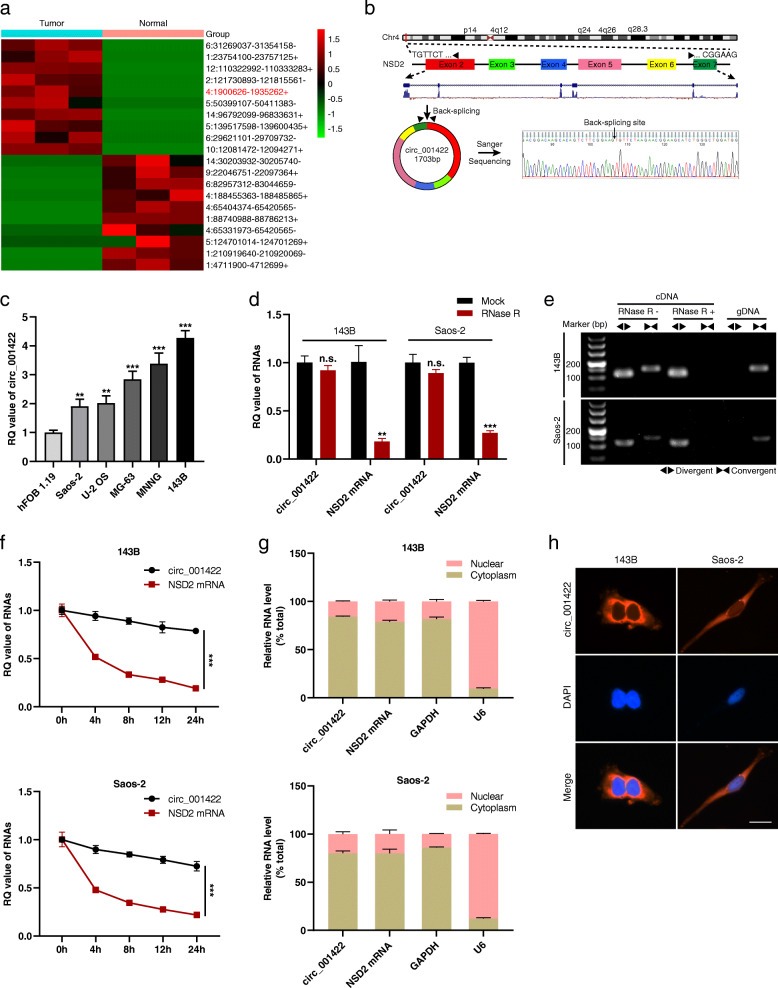
Identification and characterization of circ_001422 in OS. **a**. Cluster heatmap showing the top ten most upregulated and downregulated circRNAs identified by high-throughput sequencing in 3 paired OS tissues and adjacent noncancerous tissues. The rows show circRNAs, while the columns show samples. The red and green strips indicate upregulated and downregulated circRNAs, respectively. The list of the circRNAs is presented as Chr:5′ nt position-3′ nt position and the type of the strand (positive or negative). **b**. Schematic illustration of circ_001422 formation and the results of Sanger sequencing. **c**. Relative quantification (RQ) value of circ_001422 in OS cells and normal osteoblasts. **d**. RQ values of circ_001422 and NSD2 mRNA in OS cells under RNase R treatment. **e**. The linear and back-splicing products were amplified with convergent and divergent primers, treated with and without RNase R, and subjected to PCR. **f**. RQ values of circ_001422 and NSD2 mRNA after treatment with actinomycin D at the indicated time points. **g** & **h**. Nucleocytoplasmic fractionation and FISH revealed the subcellular localization of circ_001422 in OS cells. Nuclei were stained with DAPI, and circ_001422 probes were labeled with Cy3. Scale bar, 50 μm. n.s., not significant; ^*^P < 0.05; ^**^P < 0.01; ^***^P < 0.001

To investigate the potential involvement of circRNAs in OS, three matched OS samples and adjacent noncancerous tissues were subjected to high-throughput sequencing analysis. Based on criteria of a *P*-value < 0.05 and | fold change | ≥ 2, 374 differentially expressed candidate circRNAs were identified: 192 upregulated and 182 downregulated circRNAs in OS tissues relative to the matched noncancerous tissues. The top ten most upregulated and downregulated circRNAs are presented in the cluster heatmap (Fig. 1a). By combining these results with the circRNA annotation in the circBase database (http://www.circbase.org/), we found that circ_001422 is located on chromosome 4 (chr4: 1900626–1,935,262), has a length of 1703 bp and is generated by circularization of exons 2–7 of the host gene NSD2. The existence and structure of circ_001422 were validated through primer-specific amplification and Sanger sequencing of the PCR products (Fig. 1b). In addition, qRT-PCR further demonstrated upregulated expression of circ_001422 in OS cells relative to normal osteoblasts (Fig. 1c). Thus, circ_001422 was selected for further analyses.

CircRNAs are noncoding RNAs (ncRNAs) and are characterized by their stable structure due to the absence of a 5′ cap and a 3′ polyadenylated tail. Herein, circ_001422 and NSD2 mRNA were amplified using divergent and convergent primers, respectively. After RNase R treatment, the level of linear NSD2 mRNA decreased sharply, whereas that of circ_001422 remained stable (Fig. 1d). When both cDNA and gDNA were used as templates, NSD2 mRNA was amplified by convergent primers from both cDNA and gDNA, whereas circ_001422 was amplified by divergent primers only from cDNA (Fig. 1e). In addition, the actinomycin D-based transcriptional inhibition assay revealed that circ_001422 had a longer half-life than NSD2 mRNA (Fig. 1f). Nucleocytoplasmic fractionation and FISH further revealed that circ_001422 was localized predominantly in the cytoplasm (Fig. 1g and h).

### Correlations of circ_001422 expression with clinical characteristics

**Fig. 2:**
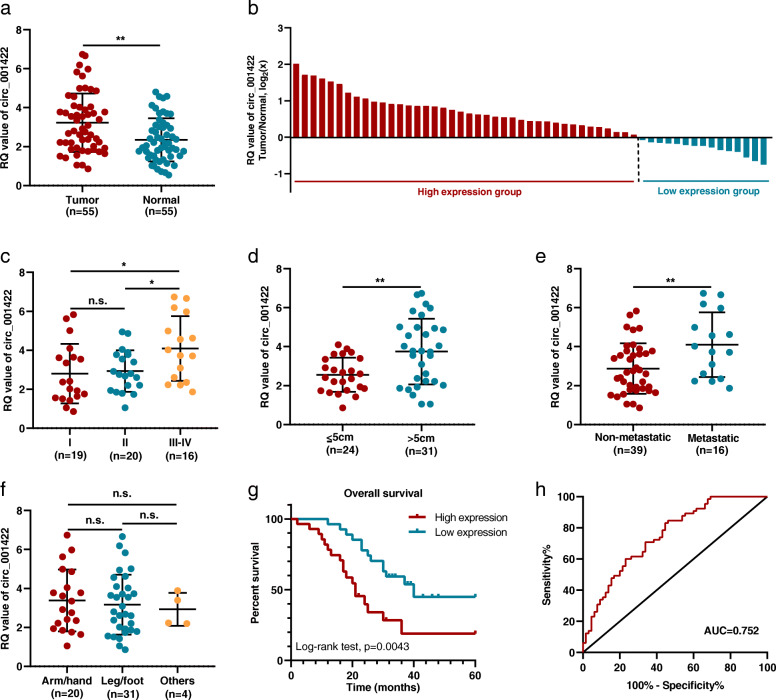
Correlation of circ_001422 expression with clinical characteristics. **a**. The relative quantification (RQ) value of circ_001422 was determined by qRT-PCR in 55 paired OS tissues and adjacent noncancerous tissues. **b**. Fold changes (log_2_) in circ_001422 expression in each paired sample, arranged in descending order. **c**. Correlation between circ_001422 expression and clinical stage. **d**. Correlation between circ_001422 expression and tumor size. **e**. Correlation between circ_001422 expression and distant metastasis. **f**. Correlation between circ_001422 expression and primary tumor location. **g**. Patients with OS were divided into two groups according to the median level of circ_001422 expression. Overall survival was analyzed by the Kaplan-Meier method with the log-rank test. **h**. ROC curve showing the diagnostic sensitivity and specificity of circ_001422 for OS. n.s., not significant; ^*^P < 0.05; ^**^P < 0.01; ^***^P < 0.001

The upregulated expression of circ_001422 was validated after analysis of OS tissues and matched noncancerous tissues from the patient cohort (*n* = 55) (Fig. 2a). In particular, overexpression of circ_001422 was observed in 40 (72.7%) of the 55 OS tissues relative to their matched noncancerous tissues (Fig. 2b). Further analyses revealed that overexpression of circ_001422 in OS tissues correlated positively with advanced clinical stage (I vs. III-IV, *P* = 0.026; II vs. III-IV, *P* = 0.048, Fig. 2c), large tumor size (*P* = 0.003, Fig. 2d) and distant metastasis (*P* = 0.005, Fig. 2e). No significant relationship was found for other parameters, including age (*P* = 0.520), sex (*P* = 0.150) and primary tumor location (*P* = 0.818, Fig. 2f) (Table 1). Kaplan-Meier survival analysis with the log-rank test was performed on OS patients in the high and low circ_001422 expression groups (based on the median circ_001422 expression level) and revealed that patients with higher circ_001422 expression levels exhibited worse overall survival rates (Fig. 2g). Moreover, receiver operating characteristic (ROC) analysis was used to determine the diagnostic potential of circ_001422 based on the 55 paired tissue samples. As shown in Fig. 2h, the area under the ROC curve (AUC) was 0.752, implying that circ_001422 is a relatively accurate diagnostic marker for OS.

### Circ_001422 promotes the proliferation and metastasis of OS cells

**Fig. 3:**
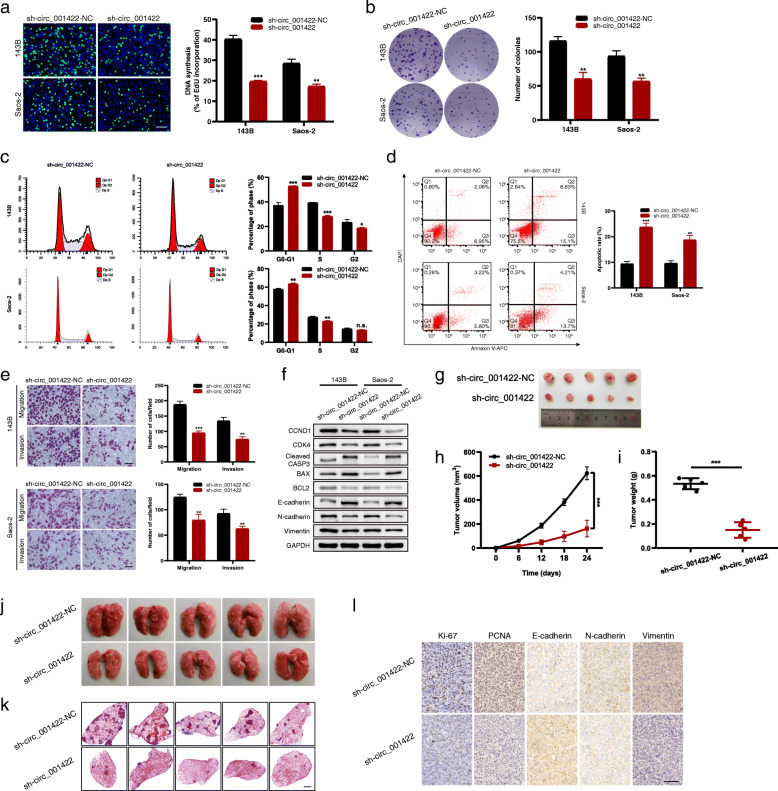
Knockdown of circ_001422 inhibits the proliferation and metastasis of OS cells. **a** & **b**. EdU incorporation (scale bar, 100 μm) and colony formation assays were performed to evaluate the effect of circ_001422 knockdown on the cell proliferation ability. **c** & **d**. Flow cytometry was performed to evaluate the effect of circ_001422 knockdown on the cell cycle distribution and apoptosis. **e**. Transwell migration and Matrigel invasion assays were performed to evaluate the effect of circ_001422 knockdown on cell migration and invasion. Scale bar, 100 μm. **f**. Western blot analysis was performed to evaluate the effect of circ_001422 knockdown on the expression of cell cycle-, apoptosis-, and EMT-associated marker proteins. **g**. Representative images of xenograft tumors in nude mice after subcutaneous implantation of 143B cells stably transduced with circ_001422 knockdown or control lentiviral vectors. **h** & **i**. Effect of circ_001422 knockdown on tumor growth and tumor weight in nude mice. **j** & **k**. Representative images of lung tissues and lung sections stained with H&E in the lung metastasis mouse model. Scale bar, 1000 μm. **l**. Tumor tissues were sectioned and subjected to immunohistochemical staining for Ki-67, PCNA, E-cadherin, N-cadherin and Vimentin. Scale bar, 100 μm. n.s., not significant; ^*^P < 0.05; ^**^P < 0.01; ^***^P < 0.001

Loss-of-function experiments were first performed by transducing 143B and Saos-2 cells with three shRNAs designed to specifically target the junction site of circ_001422. Among the shRNAs, sh-circ_001422#2 (hereafter denoted sh-circ_001422) showed the highest knockdown efficiency and was used in further analyses (Additional file 2: Fig. S1a). After knockdown of circ_001422, both the DNA synthesis and colony-forming abilities of 143B and Saos-2 cells were dramatically reduced (Fig. 3a and b). Flow cytometric analyses further revealed that circ_001422 knockdown induced G0/G1 phase arrest (Fig. 3c) and enhanced OS cell apoptosis (Fig. 3d). Moreover, the results of Transwell assays showed that circ_001422-knockdown cells had greatly decreased migration and invasion capacities (Fig. 3e). By western blot analysis (Fig. 3f), we found that circ_001422 silencing decreased the protein levels of CCND1, CDK4, BCL2, N-cadherin and Vimentin but upregulated the protein levels of cleaved CASP3, BAX and E-cadherin. Given the physiological role of these proteins, these findings suggest that circ_001422 regulates cell cycle progression, apoptosis and epithelial-mesenchymal transition (EMT), all of which impact OS development. For in vivo experiments, 143B cells transduced with stable sh-circ_001422 lentivirus or control lentivirus were injected subcutaneously (for the subcutaneous xenograft tumor model) or intravenously via the tail vein (for the lung metastasis model) into nude mice. Compared with mice in the control group, mice injected with circ_001422-knockdown cells exhibited significantly decreased tumor volumes and tumor weights (Fig. 3g-i) and a decreased number of metastatic pulmonary colonies (Fig. 3j and k). Immunohistochemical analysis further revealed that circ_001422 knockdown reduced the protein expression levels of Ki-67, PCNA, Vimentin and N-cadherin, but increased that of E-cadherin (Fig. 3l). Moreover, in the TUNEL assay more apoptotic cells were detected in the circ_001422-knockdown tumors than in the control tumors (Additional file 2: Fig. S1b).

**Fig. 4:**
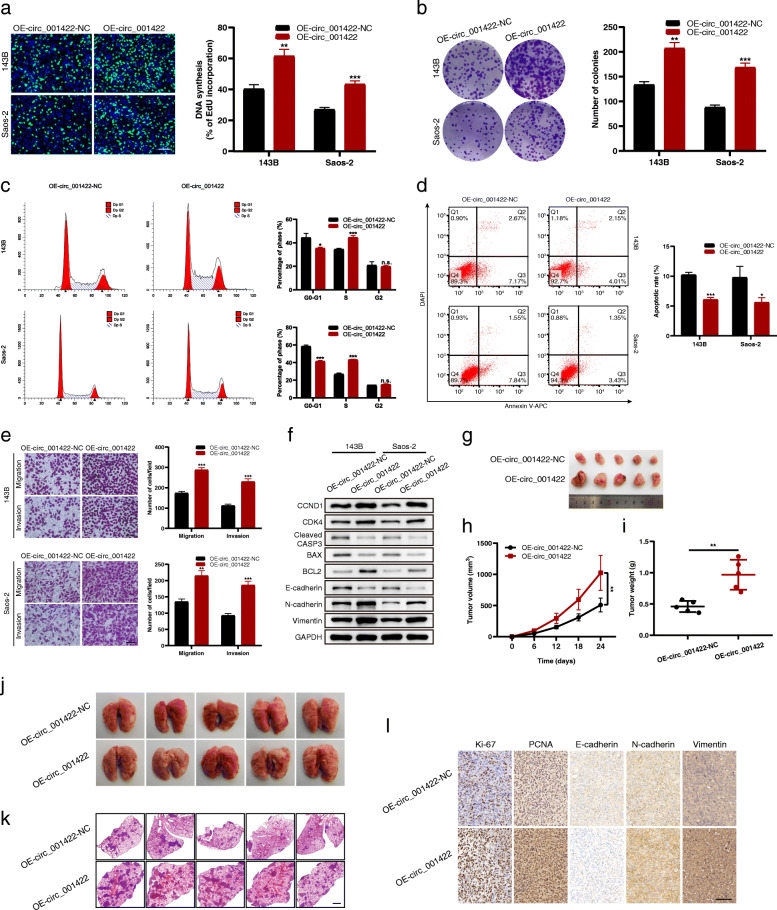
Overexpression of circ_001422 promotes the proliferation and metastasis of OS cells. **a** & **b**. EdU incorporation (scale bar, 100 μm) and colony formation assays were performed to evaluate the effect of circ_001422 overexpression on the cell proliferation ability. **c** & **d**. Flow cytometry was performed to evaluate the effect of circ_001422 overexpression on the cell cycle distribution and apoptosis. **e**. Transwell migration and Matrigel invasion assays were performed to evaluate the effect of circ_001422 overexpression on cell migration and invasion. Scale bar, 100 μm. **f**. Western blot analysis was performed to evaluate the effect of circ_001422 overexpression on the expression of cell cycle-, apoptosis-, and EMT-associated marker proteins. **g**. Representative images of xenograft tumors in nude mice after subcutaneous implantation of 143B cells stably transduced with circ_001422 overexpression or control lentiviral vectors. **h** & **i**. Effect of circ_001422 overexpression on tumor growth and tumor weight in nude mice. **j** & **k**. Representative images of lung tissues and lung sections stained with H&E in the lung metastasis mouse model. Scale bar, 1000 μm. **l**. Tumor tissues were sectioned and subjected to immunohistochemical staining for Ki-67, PCNA, E-cadherin, N-cadherin and Vimentin. Scale bar, 100 μm. n.s., not significant; ^*^P < 0.05; ^**^P < 0.01; ^***^P < 0.001

In addition, OS cell lines were transduced with the circ_001422 overexpression vector or control vector, and the overexpression efficiency was validated using qRT-PCR (Additional file 3: Fig. S2a). The results of gain-of-function experiments consistently showed that overexpression of circ_001422 markedly suppressed the apoptosis but promoted the proliferation and metastasis of OS cells (Fig. 4, Additional file 3: Fig. S2b). Collectively, these findings demonstrate that circ_001422 regulates the oncogenic and metastatic properties of OS cells.

### Circ_001422 sponges miR-195-5p

**Fig. 5:**
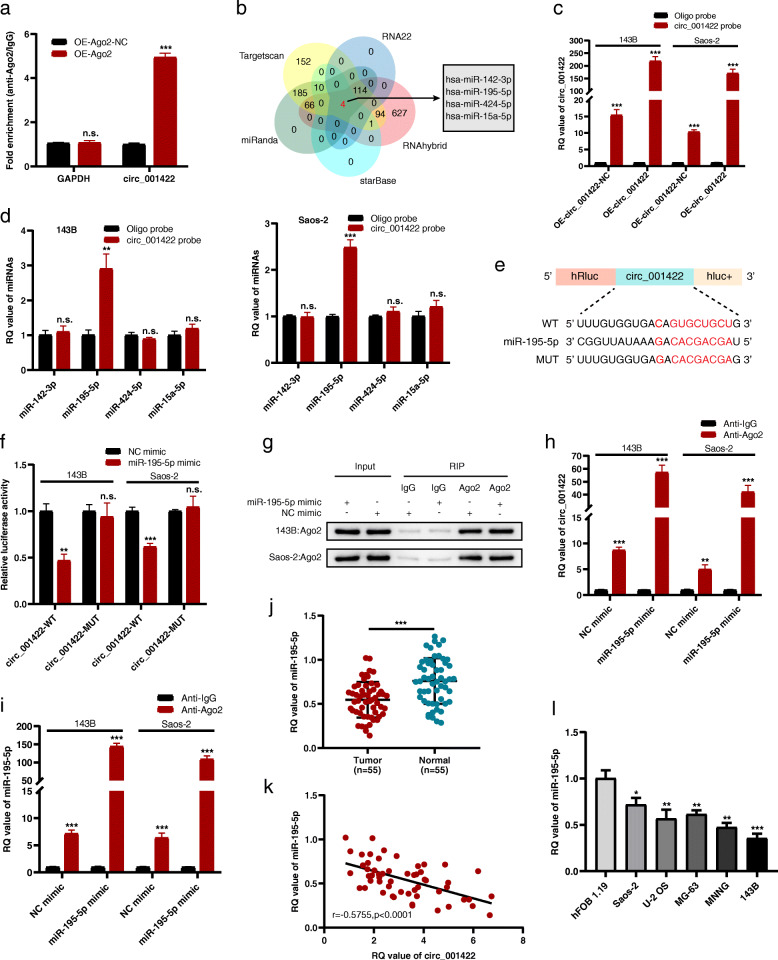
Circ_001422 sponges miR-195-5p. **a**. A RIP assay was performed to assess the circ_001422 level in 143B cells transfected with Ago2 overexpression vectors or control vectors. **b**. Schematic illustration showing the overlapping target miRNAs of circ_001422 predicted by TargetScan, miRanda, starBase, RNAhybrid and RNA22. **c**. Lysates prepared from OS cells transfected with circ_001422 overexpression vectors or control vectors were subjected to an RNA pulldown assay, and the pulldown efficiency was confirmed by qRT-PCR. **d**. The relative quantification (RQ) values of 4 candidate miRNAs in OS cell lysates were examined by qRT-PCR. **e**. The putative binding sites between miR-195-5p and circ_001422. **f**. Luciferase activity was assessed in OS cells after cotransfection with circ_001422-WT or circ_001422-MUT and the miR-195-5p mimic or NC mimic. **g**-**i**. An anti-Ago2 RIP assay was conducted in OS cells after transfection with the miR-195-5p mimic or NC mimic prior to western blot and qRT-PCR analyses to measure the expression levels of Ago2, circ_001422 and miR-195-5p. **j**. The RQ value of miR-195-5p was determined by qRT-PCR in 55 paired OS tissues and adjacent noncancerous tissues. **k**. Pearson correlation analysis of circ_001422 and miR-195-5p expression in the 55 OS tissues. **l**. RQ value of miR-195-5p in OS cells and normal osteoblasts. n.s., not significant; ^*^P < 0.05; ^**^P < 0.01; ^***^P < 0.001

Accumulating studies have revealed that circRNAs contain abundant binding sites for miRNAs and thus may act as miRNA sponges. Given that circ_001422 was localized primarily and stably in the cytoplasm, we speculated that circ_001422 might be a competitive endogenous RNA. An anti-Ago2 RIP assay was performed by transfecting 143B cells with the Ago2 overexpression plasmid or the control vector. The qRT-PCR results demonstrated that endogenous circ_001422 was immunoprecipitated more effectively from the 143B cell clones with Ago2 overexpression than from the corresponding control cells (Fig. 5a). This finding suggested that circ_001422 might bind to miRNAs via the Ago2 protein. Next, 4 candidate miRNAs were identified by determining the overlap of the prediction results from 5 bioinformatics databases (TargetScan, miRanda, starBase, RNAhybrid and RNA22) (Fig. 5b). To validate our findings, we performed a pulldown assay using a biotinylated probe specific for circ_001422. As shown in Fig. 5c and d, overexpression of circ_001422 markedly enhanced the pulldown efficiency, and only miR-195-5p was substantially pulled down by the biotinylated circ_001422 probe in both Saos-2 and 143B cells. After validating the transfection efficiencies of the miR-195-5p mimic and inhibitor (Additional file 4: Fig. S3), we performed a dual-luciferase reporter assay, which demonstrated that the miR-195-5p mimic suppressed the luciferase activity of the WT circ_001422 reporter vector (circ_001422-WT vector) but not the MUT reporter vector (circ_001422-MUT vector) (Fig. 5e and f). In addition, the results of an anti-Ago2 RIP assay revealed that Ago2, circ_001422 and miR-195-5p were efficiently immunoprecipitated in the presence of anti-Ago2 but not anti-IgG antibodies. Moreover, circ_001422 and miR-195-5p were significantly enriched in 143B and Saos-2 cells transfected with the miR-195-5p mimic compared to those transfected with the NC mimic (Fig. 5g-i). qRT-PCR further revealed exceptionally low levels of miR-195-5p in the 55 OS samples relative to the corresponding paired noncancerous tissues, and miR-195-5p expression was inversely related to circ_001422 expression (Fig. 5j and k). Furthermore, miR-195-5p expression was downregulated in OS cells compared to normal osteoblasts (Fig. 5l).

### The pro-oncogenic effect of circ_001422 depends on miR-195-5p

**Fig. 6:**
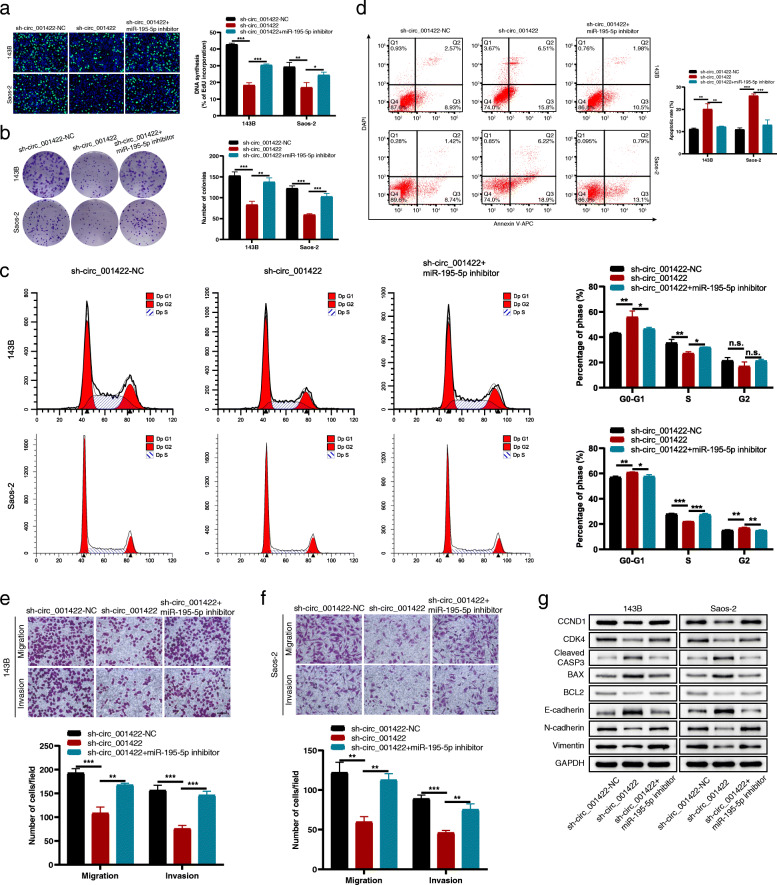
Knockdown of miR-195-5p rescues the sh-circ_001422-mediated inhibitory effects on OS cell proliferation and metastasis. **a** & **b**. OS cells were transfected with sh-circ_001422-NC or sh-circ_001422 or were cotransfected with sh-circ_001422 and the miR-195-5p inhibitor. EdU incorporation (scale bar, 100 μm) and colony formation assays were performed to evaluate the proliferation ability of OS cells in each group. **c** & **d**. Flow cytometry was used to evaluate the effect of miR-195-5p silencing on the sh-circ_001422-mediated effects on the cell cycle distribution and apoptosis. **e** & **f**. Transwell migration and Matrigel invasion assays were performed to evaluate the effect of miR-195-5p silencing on the sh-circ_001422-mediated reductions in the migration and invasion abilities. Scale bar, 100 μm. **g**. The protein levels of CCND1, CDK4, cleaved CASP3, BAX, BCL2, E-cadherin, N-cadherin and Vimentin were analyzed by western blotting and normalized to the level of GAPDH. n.s., not significant; ^*^P < 0.05; ^**^P < 0.01; ^***^P < 0.001

To determine whether circ_001422 promotes the malignant phenotype of OS cells by targeting miR-195-5p, functional rescue experiments were performed. The miR-195-5p inhibitor was first transfected into circ_001422-knockdown cells. In the EdU incorporation and colony formation assays, circ_001422 knockdown dramatically decreased the number of EdU-positive cells and inhibited colony formation in both 143B and Saos-2 cells, whereas miR-195-5p silencing reversed these effects (Fig. 6a and b). Flow cytometric analyses revealed that reintroduction of the miR-195-5p inhibitor abolished the circ_001422 knockdown-induced increases in the proportions of G0/G1 phase and apoptotic cells (Fig. 6c and d). The suppressive effects of circ_001422 knockdown on the migration and invasion of OS cells were effectively weakened by exogenous inhibition of miR-195-5p expression (Fig. 6e and f). Western blot analysis further validated the downregulated expression of cell cycle-associated proteins (CCND1 and CDK4), an antiapoptotic protein (BCL2) and mesenchymal proteins (N-cadherin and Vimentin) after circ_001422 knockdown, but miR-195-5p inhibition restored the normal expression of these proteins. In contrast, miR-195-5p knockdown markedly reversed the sh-circ_001422-mediated upregulation of proapoptotic proteins (cleaved CASP3 and BAX) and an epithelial cell marker (E-cadherin) (Fig. 6g). Furthermore, the results of functional rescue experiments revealed that the miR-195-5p mimic markedly attenuated the promotive effects of circ_001422 on cell proliferation and metastasis (Additional file 5: Fig. S4). Collectively, these findings demonstrate that circ_001422 enhances the malignant phenotypes of OS cells at least partially by sponging miR-195-5p.

### Circ_001422 positively regulates FGF2 expression in OS cells by sponging miR-195-5p

**Fig. 7:**
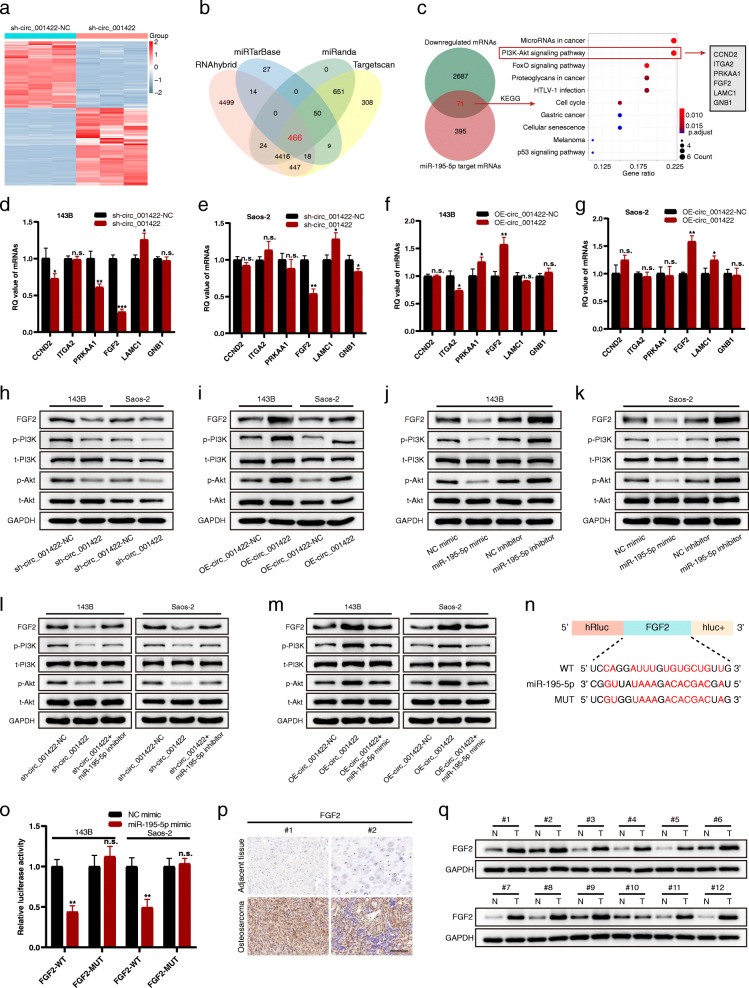
Circ_001422 positively regulates FGF2 expression in OS cells by sponging miR-195-5p. **a**. Cluster heatmap showing the significantly differentially expressed mRNAs between 143B cells transfected with sh-circ_001422 and 143B cells transfected with sh-circ_001422-NC. **b**. Venn diagram showing the overlapping target mRNAs of miR-195-5p predicted by TargetScan, miRanda, RNAhybrid and miRTarBase. **c**. The overlapping mRNAs between the downregulated mRNAs and miR-195-5p target mRNAs were subjected to KEGG enrichment analysis. **d** & **e**. qRT-PCR showed the relative quantification (RQ) values of 6 candidate genes in OS cells transfected with sh-circ_001422 or sh-circ_001422-NC. **f** & **g**. qRT-PCR showed the RQ values of 6 candidate genes in OS cells transfected with OE-circ_001422 or OE-circ_001422-NC. **h** & **i**. Western blot analysis showed the protein levels of FGF2, p-PI3K, total PI3K (t-PI3K), p-Akt and total Akt (t-Akt) in OS cells transfected with sh-circ_001422 or OE-circ_001422 or the corresponding negative control. **j** & **k**. Western blot analysis showed the protein levels of FGF2, p-PI3K, t-PI3K, p-Akt and t-Akt in OS cells transfected with the miR-195-5p mimic or miR-195-5p inhibitor or the corresponding negative control. **l** & **m**. Western blot analysis was performed to evaluate the effect of miR-195-5p silencing (or miR-195-5p overexpression) on sh-circ_001422 (or OE-circ_001422)-mediated changes in the protein levels of FGF2, p-PI3K, t-PI3K, p-Akt and t-Akt. **n**. The putative binding sites between miR-195-5p and FGF2. **o**. Luciferase activity was assessed in OS cells after cotransfection with FGF2-WT or FGF2-MUT and the miR-195-5p mimic or NC mimic. **p**. Representative images of immunohistochemical staining of FGF2 in OS tissues and paired noncancerous tissues. Scale bar, 100 μm. **q**. FGF2 protein expression in 12 OS tissues (T) and paired noncancerous tissues (N). n.s., not significant; *P < 0.05; **P < 0.01; ***P < 0.001

Given the above results, we further explored probable downstream molecules regulated by the circ_001422/miR-195-5p axis. Prior evidence demonstrates that circRNAs function as miRNA sponges to terminate the regulatory effects of miRNAs on target genes. In addition, the interaction between a miRNA and its target mRNA usually leads to degradation of the mRNA or posttranslational inhibition of gene expression. Thus, we hypothesized that the expression of circ_001422 is positively related to that of its target genes. To test this hypothesis, we first analyzed mRNA expression profiles by sequencing the differentially expressed genes (DEGs) in 143B cells with or without circ_001422 knockdown. A total of 2758 downregulated DEGs were identified based on a *P*-value < 0.05 and fold change ≤ − 2 (Fig. 7a). Further bioinformatic analysis of four databases (TargetScan, miRanda, RNAhybrid and miRTarBase) revealed 466 potential miR-195-5p target genes (Fig. 7b). After determining the overlap of the downregulated genes identified by mRNA-seq (*n* = 2758) and the potential miR-195-5p target genes predicted by the bioinformatics databases (*n* = 466), we identified 71 overlapping genes and further subjected these genes to Kyoto Encyclopedia of Genes and Genomes (KEGG) pathway enrichment analysis. As shown in Fig. 7c, “PI3K/Akt signaling pathway” was the most enriched pathway after “MicroRNAs in cancer”. All genes included in “PI3K/Akt signaling pathway” (CCND2, ITGA2, PRKAA1, FGF2, LAMC1 and GNB1) were considered candidate mRNAs potentially regulated by the circ_001422/miR-195-5p axis.

Subsequently, the levels of these 6 candidate mRNAs in 143B and Saos-2 cells were determined using qRT-PCR. Circ_001422 knockdown markedly altered the mRNA levels of FGF2 and LAMC1 in both 143B and Saos-2 cells compared to the corresponding control cells (Fig. 7d and e). After introduction of exogenous circ_001422, both 143B and Saos-2 OS cells exhibited a significant increase in FGF2 mRNA, whereas no significant change in LAMC1 mRNA was detected in 143B cells (Fig. 7f and g). Interestingly, FGF2 has been widely identified as an oncogene that participates in tumorigenesis and metastasis in multiple cancer types [23–25]. Thus, we presumed that circ_001422 might contribute to the malignant progression of OS by protecting FGF2 against degradation induced by miR-195-5p.

The western blot analysis results indicated that circ_001422 silencing effectively reduced the FGF2 protein level and inhibited the phosphorylation of PI3K and Akt (Fig. 7h). In contrast, overexpression of circ_001422 significantly enhanced FGF2 expression and PI3K/Akt signaling pathway activity (Fig. 7i). Additionally, introduction of the exogenous miR-195-5p mimic into 143B and Saos-2 cells decreased the protein levels of FGF2, p-PI3K and p-Akt. Conversely, miR-195-5p silencing exerted the opposite effects in both cell lines (Fig. 7j and k). As expected, the circ_001422-mediated promotive effect on FGF2/PI3K/Akt axis activity was attenuated after introduction of miR-195-5p (Fig. 7l and m). Next, the putative miR-195-5p binding sites in FGF2 were identified (Fig. 7n). Transfection of the FGF2-WT reporter vector with the miR-195-5p mimic but not the scrambled oligonucleotide sequence dramatically decreased luciferase activity. Mutation of the binding sequence, however, abolished the disruption of luciferase activity (Fig. 7o). The expression levels of FGF2 mRNA and the corresponding protein were increased in OS tissues compared with the corresponding noncancerous tissues (Fig. 7p and q, Additional file 6: Fig. S5a). In addition, FGF2 expression showed significant positive and negative correlations with circ_001422 expression and miR-195-5p expression, respectively, in OS tissues (Additional file 6: Fig. S5b and S5c).

### Circ_001422 performs its function via the miR-195-5p/FGF2 axis

**Fig. 8:**
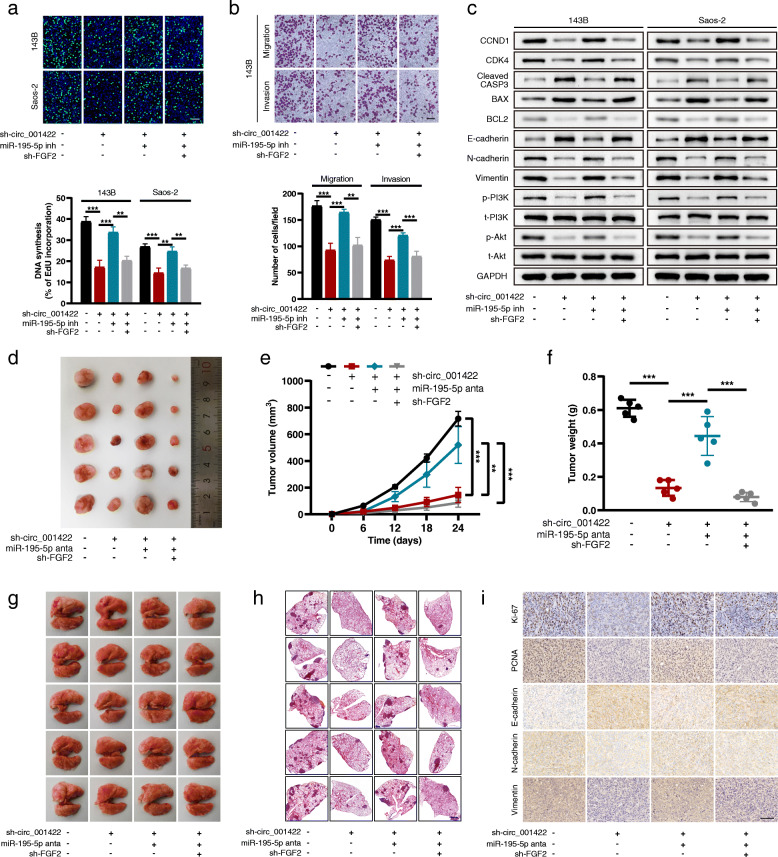
Loss-of-function experiments confirm the involvement of the circ_001422/miR-195-5p/FGF2 axis in OS progression and metastasis. **a**. Stable sh-circ_001422 OS cells were transfected with the miR-195-5p inhibitor (inh) or cotransfected with the miR-195-5p inhibitor and sh-FGF2. An EdU incorporation (scale bar, 100 μm) assay was used to evaluate the effect of circ_001422/miR-195-5p/FGF2 axis modulation on cell proliferation. **b**. Transwell migration and Matrigel invasion assays were performed to evaluate the effect of circ_001422/miR-195-5p/FGF2 axis modulation on the migration and invasion abilities of 143B cells. Scale bar, 100 μm. **c**. The protein levels of CCND1, CDK4, cleaved CASP3, BAX, BCL2, E-cadherin, N-cadherin, Vimentin, p-PI3K, t-PI3K, p-Akt and t-Akt were analyzed by western blotting and normalized to the level of GAPDH. **d**. Nude mice were distributed into 4 groups: control, sh-circ_001422, sh-circ_001422 + miR-195-5p antagomir (anta), and sh-circ_001422 + miR-195-5p antagomir + sh-FGF2. Representative images of xenograft tumors in the 4 groups are shown. **e** & **f**. Effect of circ_001422/miR-195-5p/FGF2 axis modulation on tumor growth and tumor weight in nude mice. **g** & **h**. Representative images of lung tissues and lung sections stained with H&E in the lung metastasis mouse model. Scale bar, 1000 μm. **i**. Detection of Ki-67, PCNA, E-cadherin, N-cadherin and Vimentin expression in tumor samples from nude mice in the 4 groups by IHC. Scale bar, 100 μm. n.s., not significant; ^*^P < 0.05; ^**^P < 0.01; ^***^P < 0.001

**Fig. 9:**
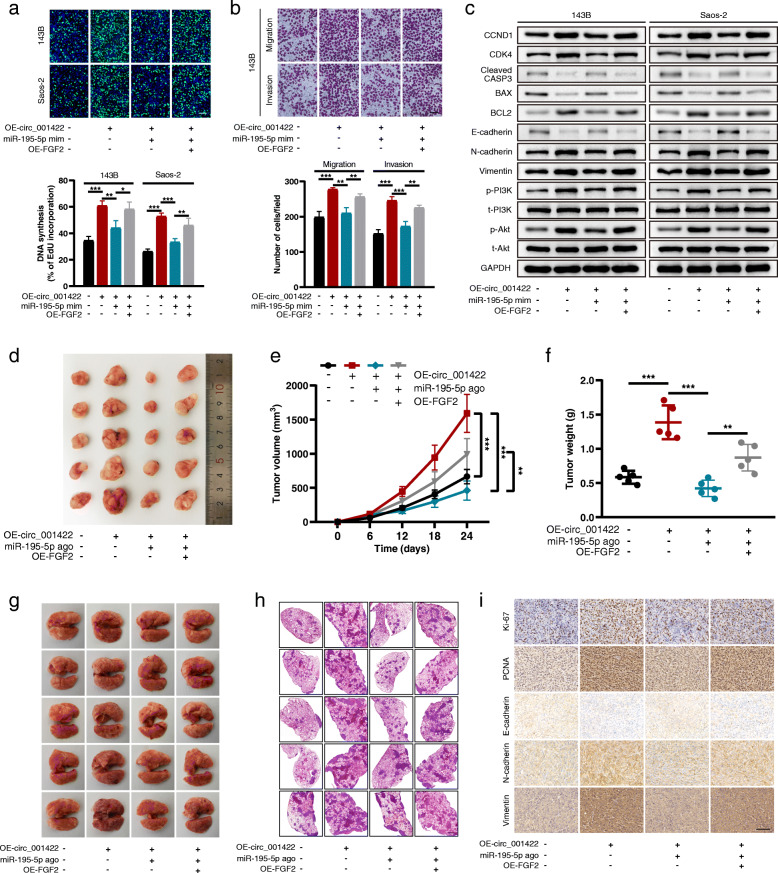
Gain-of-function experiments confirm the involvement of the circ_001422/miR-195-5p/FGF2 axis in OS progression and metastasis. **a**. Stable OE-circ_001422 OS cells were transfected with the miR-195-5p mimic (mim) or cotransfected with the miR-195-5p mimic and OE-FGF2. An EdU incorporation (scale bar, 100 μm) assay was used to evaluate the effect of circ_001422/miR-195-5p/FGF2 axis modulation on cell proliferation. **b**. Transwell migration and Matrigel invasion assays were performed to evaluate the effect of circ_001422/miR-195-5p/FGF2 axis modulation on the migration and invasion abilities of 143B cells. Scale bar, 100 μm. **c**. The protein levels of CCND1, CDK4, cleaved CASP3, BAX, BCL2, E-cadherin, N-cadherin, Vimentin, p-PI3K, t-PI3K, p-Akt and t-Akt were analyzed by western blotting and normalized to the level of GAPDH. **d**. Nude mice were distributed into 4 groups: control, OE-circ_001422, OE-circ_001422 + miR-195-5p agomir (ago), and OE-circ_001422 + miR-195-5p agomir + OE-FGF2. Representative images of xenograft tumors in the 4 groups are shown. **e** & **f**. Effect of circ_001422/miR-195-5p/FGF2 axis modulation on tumor growth and tumor weight in nude mice. **g** & **h**. Representative images of lung tissues and lung sections stained with H&E in the lung modulation mouse model. Scale bar, 1000 μm. **i**. Detection of Ki-67, PCNA, E-cadherin, N-cadherin and Vimentin expression in tumor samples from nude mice in the 4 groups by IHC. Scale bar, 100 μm. n.s., not significant; ^*^P < 0.05; ^**^P < 0.01; ^***^P < 0.001

**Fig. 10:**
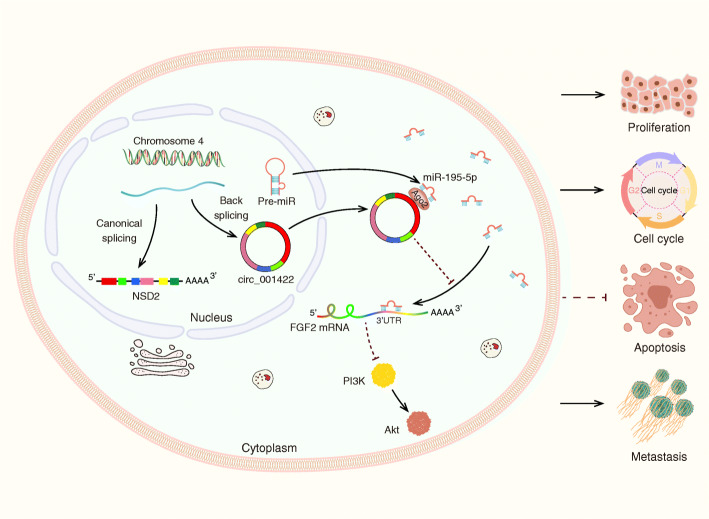
Schematic illustrating the biological role of the circ_001422/miR-195-5p/FGF2 axis in OS progression and metastasis

We performed rescue experiments to explore whether circ_001422 promotes the malignant phenotypes of OS cells by competitively interacting with miR-195-5p and then upregulating the expression of FGF2. The FGF2 overexpression and knockdown efficiencies were verified by qRT-PCR (Additional file 7: Fig. S6a and S6b). First, cells with stable circ_001422 silencing were transfected with the miR-195-5p inhibitor alone or in combination with sh-FGF2. The results of the EdU assay were consistent with previous data showing that the reduced proliferation ability of stable sh-circ_001422 cells was significantly attenuated by the miR-195-5p inhibitor but this rescue effect of the miR-195-5p inhibitor on stable sh-circ_001422 cells was effectively reversed by cotransfection with sh-FGF2 (Fig. 8a). Additionally, miR-195-5p silencing abolished circ_001422 knockdown-induced cell cycle arrest and apoptosis, while reintroduction of sh-FGF2 successfully attenuated these effects of miR-195-5p silencing (Additional file 7: Fig. S6c and S6d). The Transwell assay results revealed that the inhibitory effects of circ_001422 depletion on the cell migration and invasion capacities were dramatically weakened through exogenous inhibition of miR-195-5p. However, in cells with stable circ_001422 silencing, the suppressive effects of sh-circ_001422 on cell motility were restored after cotransfection with the miR-195-5p inhibitor and sh-FGF2 (Fig. 8b, Additional file 7: Fig. S6e). Similar trends were observed for the regulatory effects of the circ_001422/miR-195-5p/FGF2 axis on the expression of cell cycle-, apoptosis-, EMT- and PI3K/Akt signaling pathway-associated proteins (Fig. 8c). In the in vivo experiments, circ_001422 deficiency decreased subcutaneous tumor formation and lung metastases relative to those in mice injected with control cells. In addition, administration of the miR-195-5p antagomir abolished the effects of circ_001422 silencing and accelerated the growth and metastasis of OS cells, while knockdown of FGF2 reversed the pro-oncogenic role of the miR-195-5p antagomir (Fig. 8d-h). The results of immunohistochemical analysis (Fig. 8i) and TUNEL assays (Additional file 7: Fig. S6f) further validated the role of the circ_001422/miR-195-5p/FGF2 axis in OS. Moreover, functional rescue experiments were performed in cell lines with stable circ_001422 overexpression. The results, as shown in Fig. 9 and Additional file 8: Fig. S7, revealed that circ_001422 competitively interacted with miR-195-5p to upregulate FGF2 expression and activate the PI3K/Akt signaling pathway, thus facilitating the metastatic and proliferative abilities of OS cells. A schematic diagram was generated to visualize the role of the circ_001422/miR-195-5p/FGF2 axis in OS (Fig. 10).

## Discussion

OS is the most prevalent malignant bone tumor. It is highly metastatic, resulting in a very poor survival rate [2]. Approximately 80% of OS patients exhibit subclinical pulmonary micrometastases at the time of diagnosis [26]. The lack of accurate biomarkers has further hindered efforts to improve the clinical outcome of OS. Recently, the dysregulation of ncRNAs in OS has generated significant interest from the scientific community. Using global miRNA microarrays, Duan et al. analyzed the miRNA expression profiles of drug-resistant and non-drug-resistant OS cells and revealed that miR-15b inhibitors might contribute to the treatment of drug-resistant OS when coadministered with doxorubicin [27]. A separate study showed that upregulation of the long noncoding RNA (lncRNA) TP73-AS1 was closely related to advanced Enneking stage, adverse pathological features and distant metastasis in OS [28]. Unlike these two kinds of ncRNAs, circRNAs have emerged as more reliable and promising tumor biomarkers owing to their exceptionally stable structure. Advanced genome sequencing techniques have validated the roles of circRNAs in multiple cancers, including hepatocellular carcinoma [29], gastric cancer [30], colorectal cancer [31] and lung squamous cell carcinoma [32]. However, to date, the expression profiles and roles of circRNAs in OS are not well understood.

This study provides the first evidence that circ_001422 contributes to the malignant progression of OS. CircRNAs are widely accepted to be an unorthodox RNA species generated by alternative splicing of pre-mRNAs [33]. There are three main classes of circRNAs: exonic circRNAs, exon-intron circRNAs and intronic circRNAs [34]. Sanger sequencing revealed that circ_001422 is generated via back-splicing and covalent bonding of the 3′ and 5′ ends of exons 2–7 of NSD2. Interestingly, research has shown that NSD2 is an important oncogene that drives the development of multiple cancers by catalyzing histone-lysine methylation and disrupting chromatin integrity [35–37]. Additionally, linear NSD2 regulates EMT and the protein expression of BCL2 and SOX2, which facilitates cell survival, metastasis, and chemoresistance in OS [38, 39]. Herein, we revealed upregulated expression of circ_001422 in OS tissues and cells using high-throughput sequencing and qRT-PCR. Analysis of the clinicopathological characteristics of 55 OS patients revealed that circ_001422 expression positively correlated with advanced clinical stage, tumor size and distant metastasis. Functional analyses further validated the role of circ_001422 in not only promoting the proliferation and metastasis of OS cells but also modulating the apoptosis of these cells both in vivo and in vitro. These findings highlight the significant relationship between the alternatively spliced forms of the NSD2 transcript and undesirable aspects of OS.

The subcellular distribution of RNAs is intimately tied to their biological functions [40]. Accumulating evidence shows that cytoplasmic circRNAs sponge miRNAs, which represses the translation or induces the degradation of the target mRNAs after binding of the Ago2 protein [41, 42]. Herein, we found that circ_001422 is mainly a cytoplasmic RNA in OS cells. In addition, circ_001422 can recognize and bind the Ago2 protein, suggesting that circ_001422 might exert its regulatory functions via the classical method of binding to miRNAs. Among the 4 candidate miRNAs predicted by the bioinformatics databases, only miR-195-5p was further validated to exhibit a high binding capacity for circ_001422. Despite this new finding, the involvement of miR-195-5p in the pathogenesis of multiple tumors is not a new phenomenon [43, 44]. Reports on the interactions between miR-195-5p and circRNAs in cancer are scarce. Herein, we found that miR-195-5p expression was markedly decreased in clinical OS tissue samples and was inversely correlated with circ_001422 expression. Functional rescue experiments further revealed that the miR-195-5p inhibitor substantially reversed the suppressive effects of circ_001422 depletion on OS cell proliferation and metastasis, whereas the miR-195-5p mimic abolished the promotive effects of circ_001422 overexpression.

Moreover, we found that FGF2 is a downstream target of miR-195-5p in OS cells. Consistent with the competing endogenous RNA theory, our current study revealed a positive correlation of FGF2 expression with circ_001422 expression and a negative correlation of FGF2 expression with miR-195-5p expression in clinical OS tissues. In addition, bioinformatic analysis and functional experiments revealed that circ_001422 upregulated FGF2 expression by sponging miR-195-5p. This event also triggered PI3K/Akt pathway activation to accelerate OS progression via mechanisms including suppression of apoptosis and promotion of cell proliferation, migration and invasion. FGF2, also called bFGF, was among the first angiogenic factors identified [45]. Evidence indicates that FGF2 is implicated in diverse biological processes, including neurodevelopment, immune homeostasis, angiogenesis and neoplastic transformation [46]. Although the role of FGF2 in malignancies remains controversial, FGF2 has been proposed to act as a pro-oncogenic regulator during the development of OS [47–49]. In the present study, abnormally elevated levels of FGF2 mRNA and protein were consistently observed in OS tissues and cells. Furthermore, the PI3K/Akt pathway was verified to be involved in carcinogenesis mediated by the circ_001422/miR-195-5p/FGF2 axis in OS. The PI3K/Akt cascade controls basic intracellular processes, and abnormal activation of this pathway is quite prevalent in diverse neoplasms [50, 51].

Our study has several limitations. First, the subcutaneous xenograft and lung metastasis models used in this study may not fully mimic the natural OS microenvironment. Thus, some of our findings may not be reproducible in the natural disease state. Second, less invasive or noninvasive methods for detection of highly specific biomarkers in body fluids are more convenient and acceptable than current approaches. Indeed, previous evidence has demonstrated that some circRNAs may be stably detected by liquid biopsy [52, 53]. Thus, the expression profiles of circ_001422 in body fluids such as serum, plasma and urine warrant further investigation. Finally, we focused only on the roles of circ_001422 in tumor proliferation and metastasis. More detailed studies are necessary to explore the impact of circ_001422 on other malignant biological behaviors of OS cells, including chemoresistance, angiogenesis and immune escape.

## Conclusions

In summary, this research showed that circ_001422 promotes the progression and metastasis of OS via the miR-195-5p/FGF2/PI3K/Akt axis. Our findings elucidate a novel regulatory network that may offer new insight into the identification of potential biomarkers or therapeutic targets for OS.

## Supplementary Information

**Additional file 1: Table S1.** Primer sequences used for qRT-PCR in this study.

**Additional file 2: Figure S1.** Knockdown of circ_001422 inhibits the proliferation and metastasis of OS cells. a. The circ_001422 knockdown efficiency was verified by qRT-PCR. b. A TUNEL assay was performed to evaluate the effect of circ_001422 knockdown on apoptosis in vivo. Scale bar, 100 μm. n.s., not significant; ^*^P < 0.05; ^**^P < 0.01; ^***^P < 0.001.

**Additional file 3: Figure S2.** Overexpression of circ_001422 promotes the proliferation and metastasis of OS cells. a. The circ_001422 overexpression efficiency was verified by qRT-PCR. b. A TUNEL assay was performed to evaluate the effect of circ_001422 overexpression on apoptosis in vivo. Scale bar, 100 μm. n.s., not significant; ^*^P < 0.05; ^**^P < 0.01; ^***^P < 0.001.

**Additional file 4: Figure S3.** Circ_001422 sponges miR-195-5p. a & b The transfection efficiencies of the miR-195-5p mimic and inhibitor in OS cells were verified by qRT-PCR. n.s., not significant; ^*^P < 0.05; ^**^P < 0.01; ^***^P < 0.001.

**Additional file 5: Figure S4.** Overexpression of miR-195-5p rescues the OE-circ_001422-mediated promotive effects on OS cell proliferation and metastasis. a & b. OS cells were transfected with OE-circ_001422-NC or OE-circ_001422 or were cotransfected with OE-circ_001422 and the miR-195-5p mimic. EdU incorporation (scale bar, 100 μm) and colony formation assays were performed to evaluate the proliferation ability of OS cells in each group. c & d. Flow cytometry was performed to evaluate the effect of miR-195-5p overexpression on the OE-circ_001422-mediated effects on the cell cycle distribution and apoptosis. e & f. Transwell migration and Matrigel invasion assays were performed to evaluate the effect of miR-195-5p overexpression on the OE-circ_001422-mediated enhancement of the migration and invasion abilities. Scale bar, 100 μm. g. The protein levels of CCND1, CDK4, cleaved CASP3, BAX, BCL2, E-cadherin, N-cadherin and Vimentin were analyzed by western blotting and normalized to the level of GAPDH. n.s., not significant; ^*^P < 0.05; ^**^P < 0.01; ^***^P < 0.001.

**Additional file 6: Figure S5.** Circ_001422 positively regulates FGF2 expression in OS cells by sponging miR-195-5p. a. The relative quantification (RQ) value of FGF2 in 55 paired OS tissues and adjacent noncancerous tissues was determined by qRT-PCR. b. Pearson correlation analysis of circ_001422 and FGF2 expression in the 55 OS tissues. c. Pearson correlation analysis of miR-195-5p and FGF2 expression in the 55 OS tissues. n.s., not significant; *P < 0.05; **P < 0.01; ***P < 0.001.

**Additional file 7: Figure S6.** Loss-of-function experiments confirm the involvement of the circ_001422/miR-195-5p/FGF2 axis in OS progression and metastasis. a & b. The FGF2 knockdown and overexpression efficiencies were verified by qRT-PCR. c & d. Flow cytometry was used to evaluate the effect of circ_001422/miR-195-5p/FGF2 axis modulation on the cell cycle distribution and apoptosis. e. Transwell migration and Matrigel invasion assays were performed to evaluate the effect of circ_001422/miR-195-5p/FGF2 axis modulation on the migration and invasion abilities of Saos-2 cells. Scale bar, 100 μm. f. A TUNEL assay was performed to evaluate the effect of circ_001422/miR-195-5p/FGF2 axis modulation on apoptosis in vivo. Scale bar, 100 μm. n.s., not significant; ^*^P < 0.05; ^**^P < 0.01; ^***^P < 0.001.

**Additional file 8: Figure S7.** Gain-of-function experiments confirm the involvement the of circ_001422/miR-195-5p/FGF2 axis in OS progression and metastasis. a & b. Flow cytometry was used to evaluate the effect of circ_001422/miR-195-5p/FGF2 axis modulation on cell cycle distribution and apoptosis. c. Transwell migration and Matrigel invasion assays were performed to evaluate the effect of circ_001422/miR-195-5p/FGF2 axis modulation on the migration and invasion abilities of Saos-2 cells. Scale bar, 100 μm. d. A TUNEL assay was performed to evaluate the effect of circ_001422/miR-195-5p/FGF2 axis modulation on apoptosis in vivo. Scale bar, 100 μm. n.s., not significant; ^*^P < 0.05; ^**^P < 0.01; ^***^P < 0.001.

## Data Availability

The datasets used and/or analyzed during the current study are available from the corresponding author on reasonable request.

## Abbreviations

circRNAs: Circular RNAs
OS: Osteosarcoma
circ_001422: Hsa_circ_001422
miR-195-5p: MicroRNA-195-5p
FGF2: Fibroblast growth factor 2
PI3K: Phosphoinositide 3-kinase
Akt: Protein kinase B
ncRNAs: Noncoding RNAs
miRNAs: MicroRNAs
DMEM: Dulbecco’s modified Eagle’s medium
FBS: Fetal bovine serum
qRT-PCR: Quantitative real-time polymerase chain reaction
cDNA: Complementary DNA
GAPDH: Glyceraldehyde-3-phosphate dehydrogenase
gDNA: Genomic DNA
FISH: Fluorescence in situ hybridization
NC: Negative control
shRNAs: Short hairpin RNAs
EdU: 5-Ethynyl-2′-deoxyuridine
PBS: Phosphate-buffered saline
APC: Annexin-V-allophycocyanin
DAPI: 4′,6-Diamidino-2-phenylindole
TBST: Tris-buffered saline-Tween
CCND1: Cyclin D1
CDK4: Cyclin dependent kinase 4
CASP3: Caspase 3
BAX: B cell lymphoma/leukemia-2 associated X
BCL2: B cell lymphoma/leukemia-2
HRP: Horseradish peroxidase
H&E: Hematoxylin and eosin
IHC: Immunohistochemistry
PCNA: Proliferating cell nuclear antigen
TUNEL: Terminal deoxynucleotidyl transferase dUTP nick end labeling
RIP: RNA immunoprecipitation
Ago2: Argonaute2
WT: Wild-type
MUT: Mutant
NSD2: Nuclear receptor binding SET domain protein 2
KEGG: Kyoto Encyclopedia of Genes and Genomes
CCND2: Cyclin D2
ITGA2: Integrin subunit alpha 2
PRKAA1: Protein kinase AMP-activated catalytic subunit alpha 1
LAMC1: Laminin subunit gamma 1
GNB1: G protein subunit beta 1
EMT: Epithelial-mesenchymal transition
lncRNA: Long noncoding RNA
