# Supplementary material for: Circular RNA circ_001422 promotes the progression and metastasis of osteosarcoma via the miR-195-5p/FGF2/PI3K/Akt axis
Source: J Exp Clin Cancer Res. 2021 Jul 16;40:235. doi: 10.1186/s13046-021-02027-0 (PMC8283840; doi:10.1186/s13046-021-02027-0)
Supplement: Supplementary file 1 — Additional file 1: Table S1. Primer sequences used for qRT-PCR in this study. [file 13046_2021_2027_MOESM1_ESM.docx]

**Additional file 1:** **Table S1.** Primer sequences used for qRT-PCR in this study.

**Table S1** Primer sequences used for qRT-PCR in this study

| Gene | Forward primer (5’ to 3’) | Reverse primer (5’ to 3’) |
| --- | --- | --- |
| Circ_001422 | CAAGCACAGTCTTCGGAAGT | TTCTGGTGCCTGCTTCATCT |
| NSD2 | AGACAGATGGCAAAGGGTGG | GGGTGATGTCGTTCTCGTGT |
| miR-142-3p | GTCGTATCCAGTGCAGGG | CGACGTGTAGTGTTTCCTA |
| miR-195-5p | CGCAGCACAGAAATATTGGC | CTCAACTGGTGTCGTGGAGTC |
| miR-424-5p | GCGGCCAGCAGCAATTCATG | CAGCCACAAAAGAGCACAAT |
| miR-15a-5p | TAGCAGCACATAATGGTTTGT | GCGAGCACAGAATTAATACGAC |
| CCND2 | TGCAGAAGGACATCCAACCC | GTTGCAGATGGGACTTCGGA |
| ITGA2 | AGTGGCTTTCCTGAGAACCG | CTGGTGAGGATCAAGCCGAG |
| PRKAA1 | CCTGTGACAAGCACTTACTCC | CTAACTGATCCCGATCTCTGTG |
| FGF2 | AAAAACGGGGGCTTCTTCCT | ACGGTTAGCACACACTCCTT |
| LAMC1 | ACCGACTACAACAACCAGGC | GGTGTGGAACTTGAGACGCA |
| GNB1 | GCTGGGTACGACGACTTCAA | ATCCACATGCTACTGGCGTT |
| U6 | CTCGCTTCGGCAGCACA | AACGCTTCACGAATTTGCGT |
| GAPDH | CTCCAAAATCAAGTGGGGCG | TGGTTCACACCCATGACGAA |
