# Supplementary figures and images for: Circular RNA circ_001422 promotes the progression and metastasis of osteosarcoma via the miR-195-5p/FGF2/PI3K/Akt axis
Source: J Exp Clin Cancer Res. 2021 Jul 16;40:235. doi: 10.1186/s13046-021-02027-0 (PMC8283840; doi:10.1186/s13046-021-02027-0)

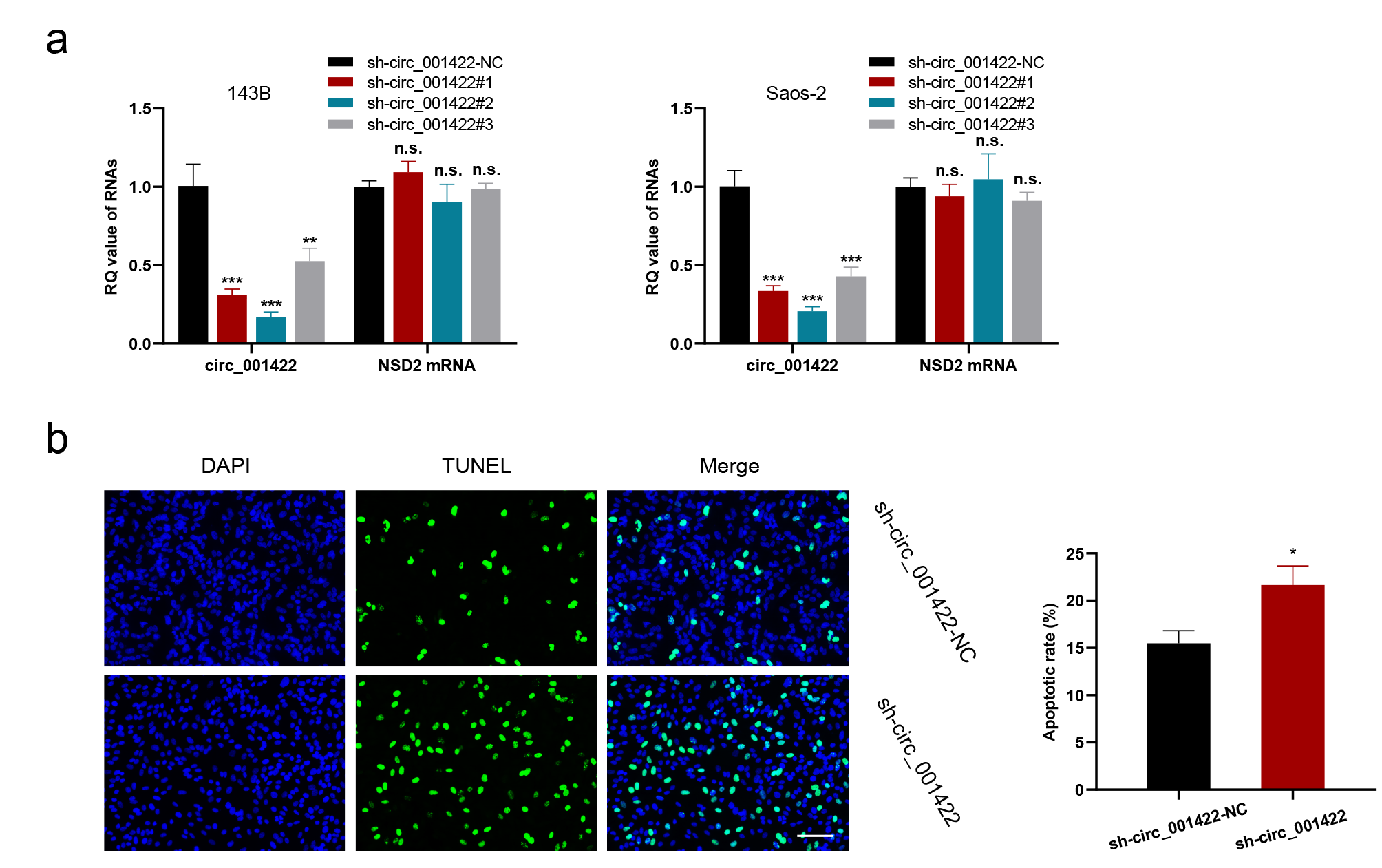

Supplement: Supplementary file 2 — Additional file 2: Figure S1. Knockdown of circ_001422 inhibits the proliferation and metastasis of OS cells. a. The circ_001422 knockdown efficiency was verified by qRT-PCR. b. A TUNEL assay was performed to evaluate the effect of circ_001422 knockdown on apoptosis in vivo. Scale bar, 100 μm. n.s., not significant; *P < 0.05; **P < 0.01; ***P < 0.001. [file 13046_2021_2027_MOESM2_ESM.tif]

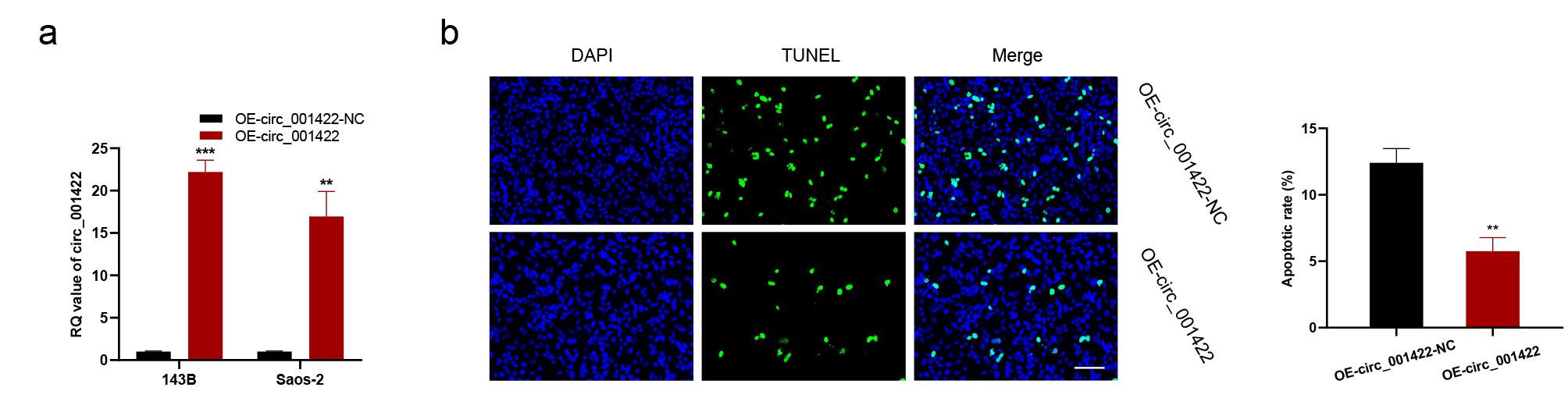

Supplement: Supplementary file 3 — Additional file 3: Figure S2. Overexpression of circ_001422 promotes the proliferation and metastasis of OS cells. a. The circ_001422 overexpression efficiency was verified by qRT-PCR. b. A TUNEL assay was performed to evaluate the effect of circ_001422 overexpression on apoptosis in vivo. Scale bar, 100 μm. n.s., not significant; *P < 0.05; **P < 0.01; ***P < 0.001. [file 13046_2021_2027_MOESM3_ESM.tif]

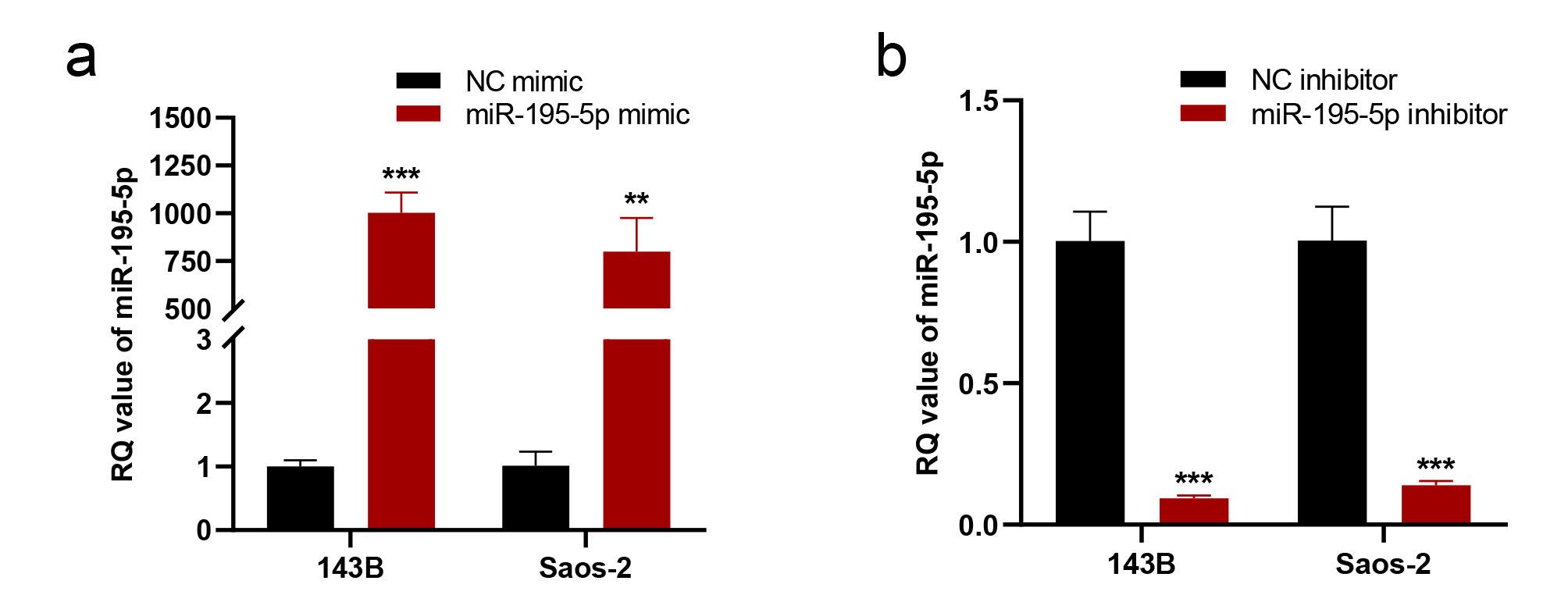

Supplement: Supplementary file 4 — Additional file 4: Figure S3. Circ_001422 sponges miR-195-5p. a & b The transfection efficiencies of the miR-195-5p mimic and inhibitor in OS cells were verified by qRT-PCR. n.s., not significant; *P < 0.05; **P < 0.01; ***P < 0.001. [file 13046_2021_2027_MOESM4_ESM.tif]

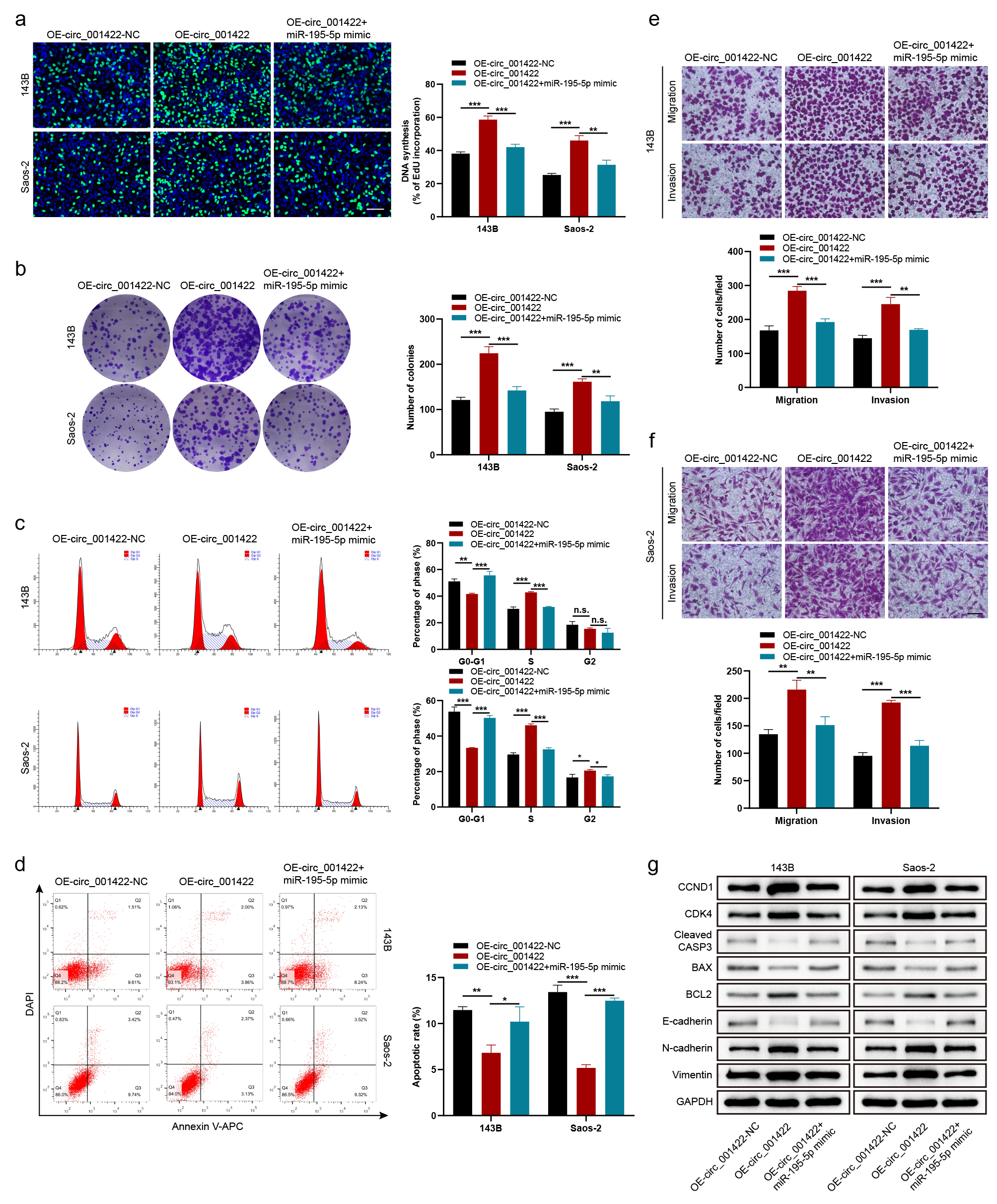

Supplement: Supplementary file 5 — Additional file 5: Figure S4. Overexpression of miR-195-5p rescues the OE-circ_001422-mediated promotive effects on OS cell proliferation and metastasis. a & b. OS cells were transfected with OE-circ_001422-NC or OE-circ_001422 or were cotransfected with OE-circ_001422 and the miR-195-5p mimic. EdU incorporation (scale bar, 100 μm) and colony formation assays were performed to evaluate the proliferation ability of OS cells in each group. c & d. Flow cytometry was performed to evaluate the effect of miR-195-5p overexpression on the OE-circ_001422-mediated effects on the cell cycle distribution and apoptosis. e & f. Transwell migration and Matrigel invasion assays were performed to evaluate the effect of miR-195-5p overexpression on the OE-circ_001422-mediated enhancement of the migration and invasion abilities. Scale bar, 100 μm. g. The protein levels of CCND1, CDK4, cleaved CASP3, BAX, BCL2, E-cadherin, N-cadherin and Vimentin were analyzed by western blotting and normalized to the level of GAPDH. n.s., not significant; *P < 0.05; **P < 0.01; ***P < 0.001. [file 13046_2021_2027_MOESM5_ESM.tif]

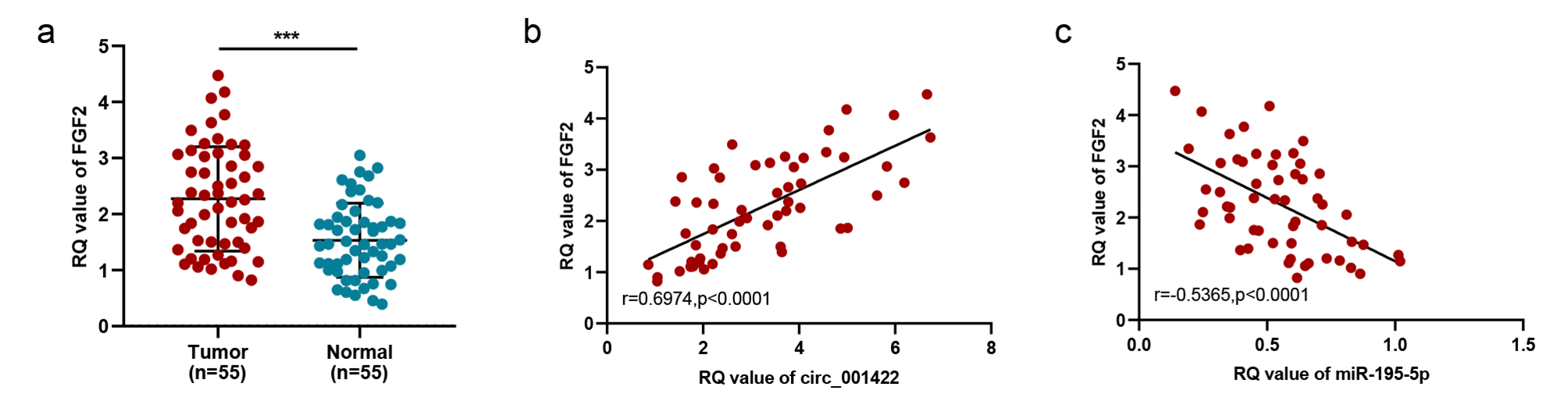

Supplement: Supplementary file 6 — Additional file 6: Figure S5. Circ_001422 positively regulates FGF2 expression in OS cells by sponging miR-195-5p. a. The relative quantification (RQ) value of FGF2 in 55 paired OS tissues and adjacent noncancerous tissues was determined by qRT-PCR. b. Pearson correlation analysis of circ_001422 and FGF2 expression in the 55 OS tissues. c. Pearson correlation analysis of miR-195-5p and FGF2 expression in the 55 OS tissues. n.s., not significant; *P < 0.05; **P < 0.01; ***P < 0.001. [file 13046_2021_2027_MOESM6_ESM.tif]

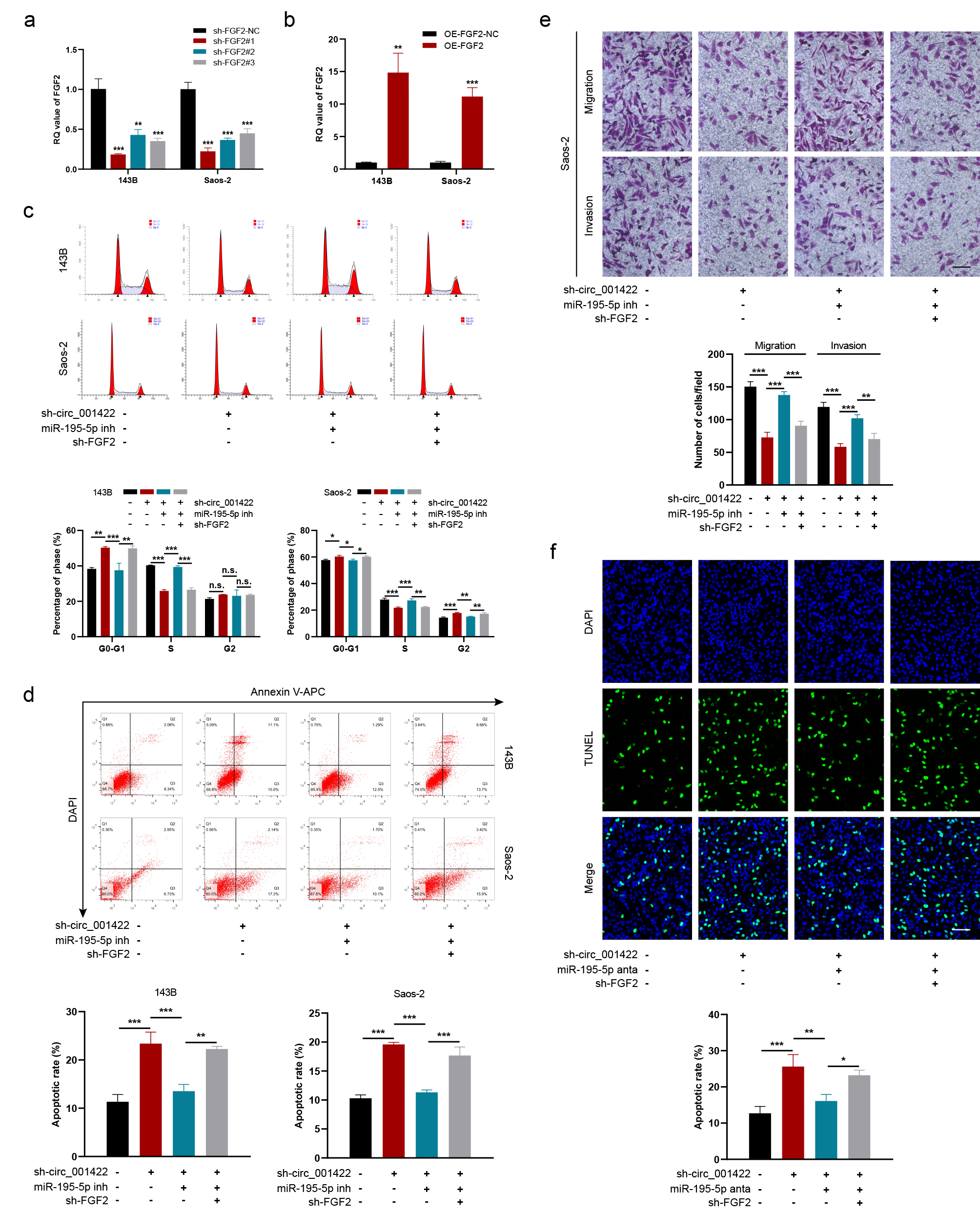

Supplement: Supplementary file 7 — Additional file 7: Figure S6. Loss-of-function experiments confirm the involvement of the circ_001422/miR-195-5p/FGF2 axis in OS progression and metastasis. a & b. The FGF2 knockdown and overexpression efficiencies were verified by qRT-PCR. c & d. Flow cytometry was used to evaluate the effect of circ_001422/miR-195-5p/FGF2 axis modulation on the cell cycle distribution and apoptosis. e. Transwell migration and Matrigel invasion assays were performed to evaluate the effect of circ_001422/miR-195-5p/FGF2 axis modulation on the migration and invasion abilities of Saos-2 cells. Scale bar, 100 μm. f. A TUNEL assay was performed to evaluate the effect of circ_001422/miR-195-5p/FGF2 axis modulation on apoptosis in vivo. Scale bar, 100 μm. n.s., not significant; *P < 0.05; **P < 0.01; ***P < 0.001. [file 13046_2021_2027_MOESM7_ESM.tif]

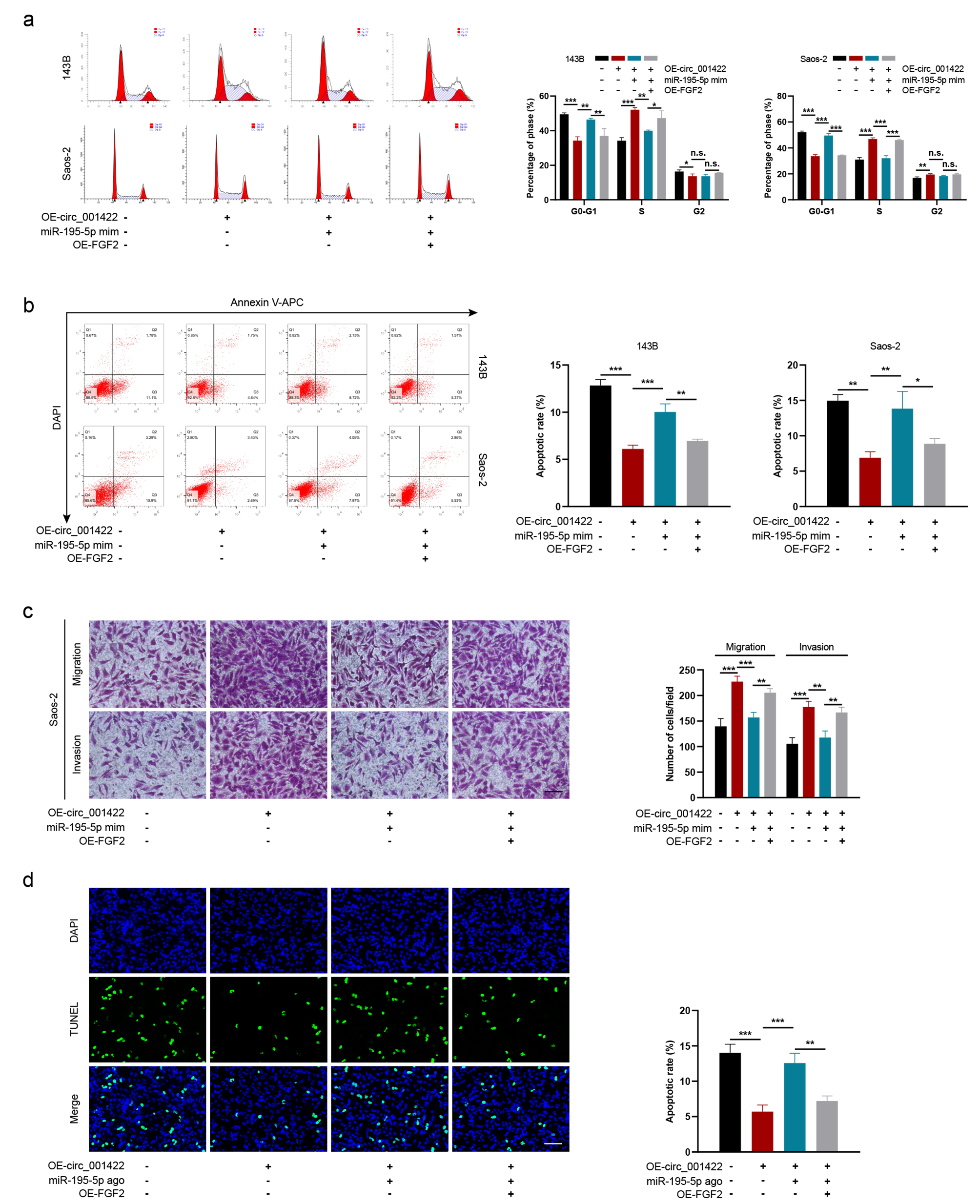

Supplement: Supplementary file 8 — Additional file 8: Figure S7. Gain-of-function experiments confirm the involvement the of circ_001422/miR-195-5p/FGF2 axis in OS progression and metastasis. a & b. Flow cytometry was used to evaluate the effect of circ_001422/miR-195-5p/FGF2 axis modulation on cell cycle distribution and apoptosis. c. Transwell migration and Matrigel invasion assays were performed to evaluate the effect of circ_001422/miR-195-5p/FGF2 axis modulation on the migration and invasion abilities of Saos-2 cells. Scale bar, 100 μm. d. A TUNEL assay was performed to evaluate the effect of circ_001422/miR-195-5p/FGF2 axis modulation on apoptosis in vivo. Scale bar, 100 μm. n.s., not significant; *P < 0.05; **P < 0.01; ***P < 0.001. [file 13046_2021_2027_MOESM8_ESM.tif]
